# Supplementary material for: Solar-driven direct air capture to produce sustainable aviation fuel
Source: Nat Commun. 2026 Jan 8;17:1223. doi: 10.1038/s41467-025-67977-x (PMC12864789; doi:10.1038/s41467-025-67977-x)
Supplement: Supplementary file 1 — Supplementary Information [file 41467_2025_67977_MOESM1_ESM.pdf]

Supplementary Information

Solar-driven direct air capture to produce sustainable aviation fuel

*Yide Han[1], Olajide Otitoju[1], Ariane D.N. Kamkeng[1], Meihong Wang[1, *], Hui Yan[2], Fisher Millard[3], Wenli Du[4,*], Feng Qian[4,*]*

[1]Department of Chemical and Biological Engineering, The University of Sheffield, Sheffield S1 3JD, UK

[2]Department of Electrical and Electronic Engineering, University of Manchester, Manchester M13 9PL, UK

[3]Net Zero Energy, AtkinsRéalis, Edinburgh, EH3 8EG, UK

[4]Key Laboratory of Advanced Control and Optimization for Chemical Process of the Ministry of Education, East China University of Science and Technology, 130 Meilong Road, Shanghai 200237, China

[*]Correspondence: meihong.wang@sheffield.ac.uk, wldu@ecust.edu.cn, fqian@ecust.edu.cn

Supplementary Note 1: Process flow information of solar-driven DACCU process

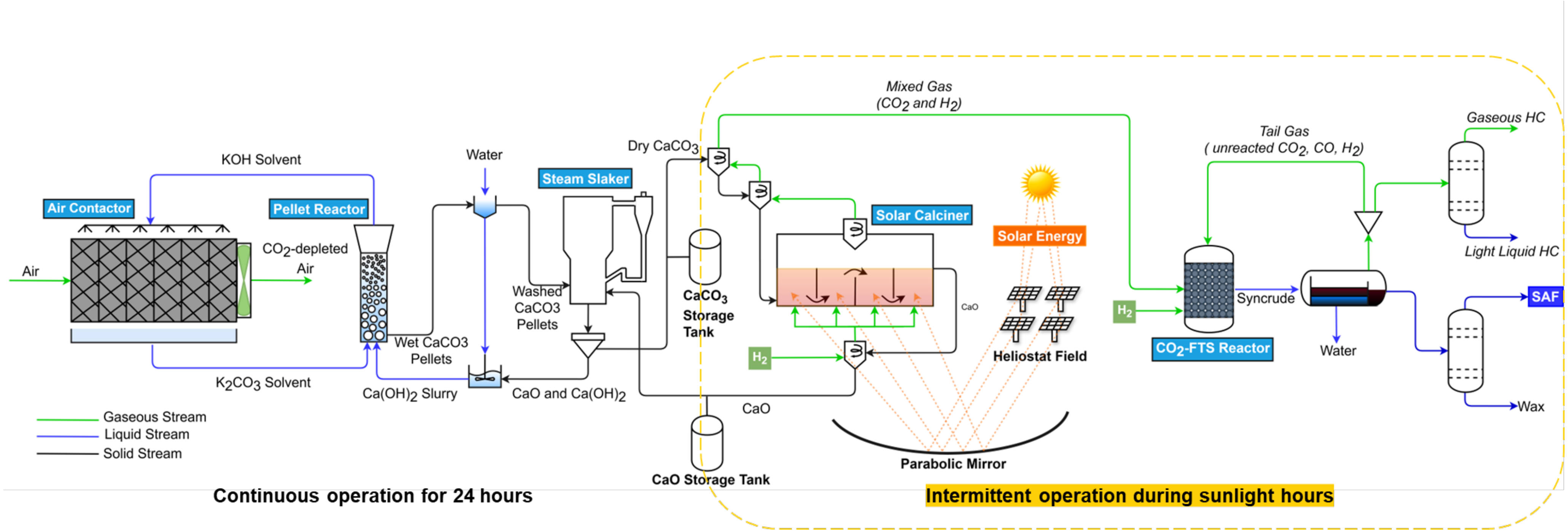

Supplementary Figure 1: Continuous and intermittent operation for the solar-driven DACCU process.

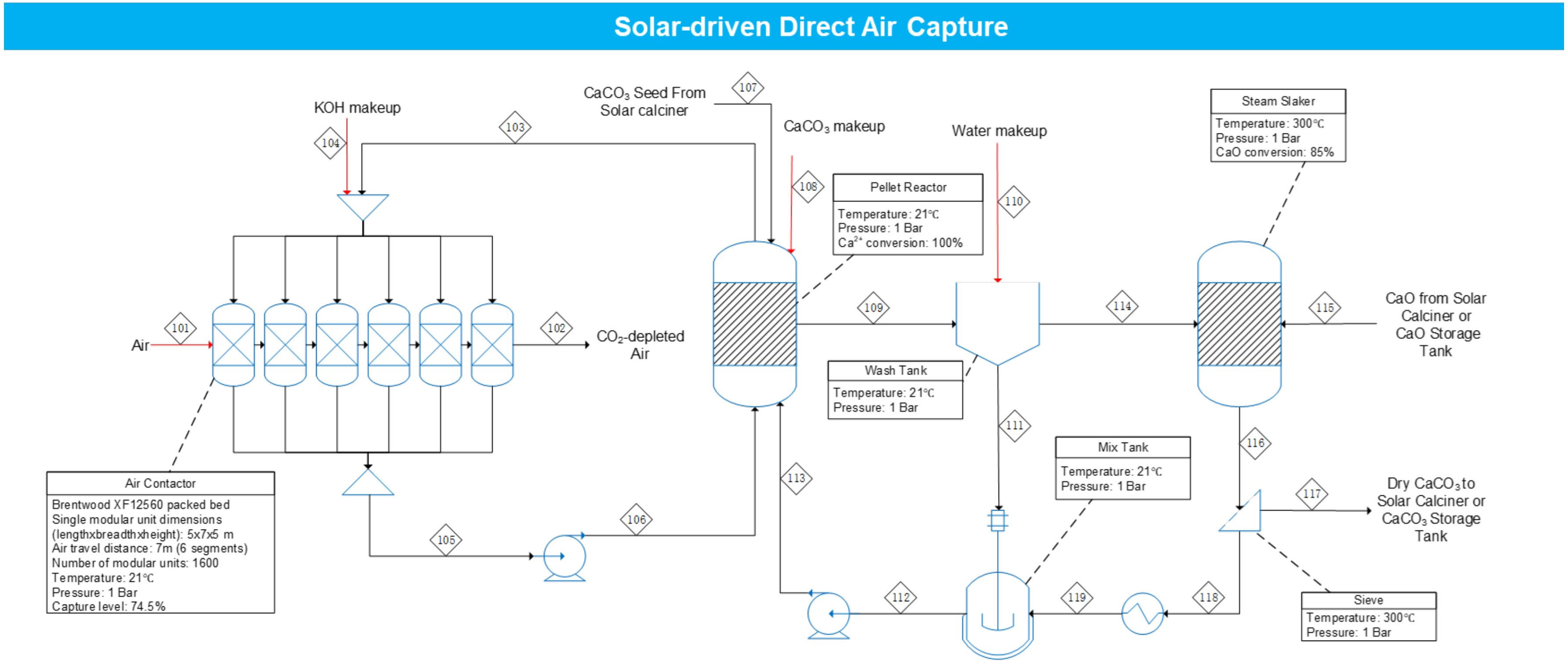

Component	Units	101	102	103	104	105	106	107	108	109	110	111	112	113	114	115	116	117	118	119
Total Flow	t/hr	251,000	251,456	32,630	**10**	32,174	32,174	14	**27**	330	516	523	716	716	323	152	479	285	194	194
Temperature	°C	21	19	21	21	18	18	21	21	21	21	21	21	21	21	450	300	300	300	85
Pressure	bar	1	1	1.4	1	1	1.8	1	1	1	1	1	1	1.8	1	1	1	1	1	1
CO_2	t/hr	160	41			0	0			0		0	0	0	0					
N_2	t/hr	190,650	190,650			0.3	0.3			0		0	0	0	0					
O_2	t/hr	57,730	57,730			0.2	0.2			0		0	0	0	0					
H_2O	t/hr	2,460	3,025	28,950		28,434	28,434			39	516	518	511	511	37					
K^+	t/hr		7	2,322		2,315	2,315			3		3	3	3	0					
OH^-	t/hr		1	556		462	462			1		1	1	1	0					
CO_3^{2-}	t/hr		3	802		962	962			1		1	1	1	0					
HCO_3^-	t/hr		0	0		0	0			0		0	0	0	0					
H_3O+	t/hr		0	0		0	0			0		0	0	0	0					
**KOH**	**t/hr**				**10**				**27**											
$Ca(OH)_2$	t/hr												201	201						
$CaCO_3$(S)	t/hr							14		285					285		285	285		
$Ca(OH)_2$(S)	t/hr																171		171	171
CaO(S)	t/hr															152	23		23	23

Supplementary Figure 2: Process flow information of the solar-driven DAC section, excluding the solar calcination.

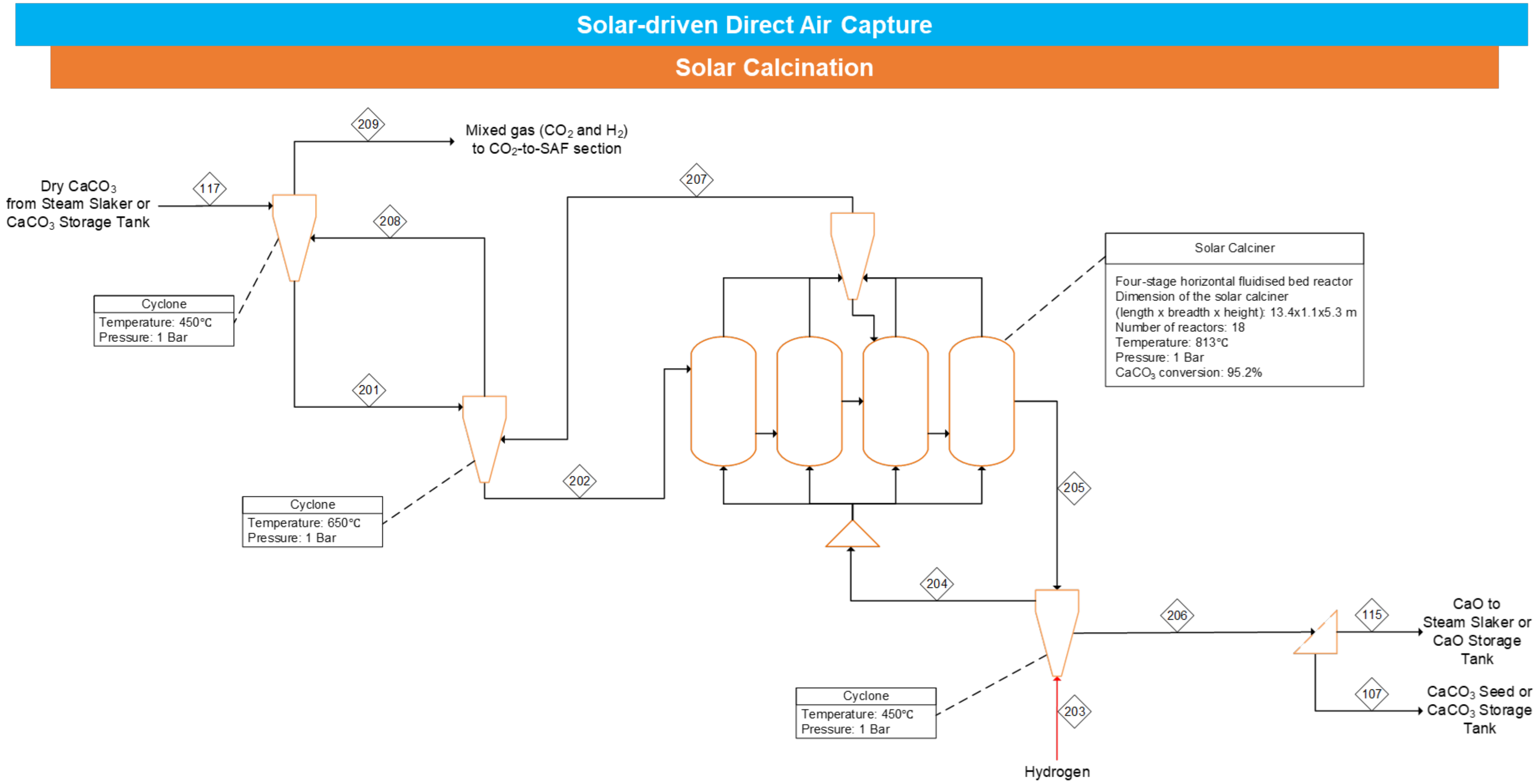

Component	Units	201	202	203	204	205	206	207	208	209
Total Flow	t/hr	855.9	855.9	27.9	27.9	497.7	497.7	386.4	386.4	386.4
Temperature	°C	486	630	21	449	813	450	813	650	450
Pressure	bar	1	1	1	1	1	1	1	1	1
CO_2	t/hr							358.2	358.2	358.2
H_2	t/hr			27.9	27.9			27.9	27.9	27.9
$CaCO_3$(S)	t/hr	855.9	855.9			41.1	41.1			
CaO(S)	t/hr					456.6	456.6			

Supplementary Figure 3: Process flow information of solar calcination subsection in solar-driven DAC section.

CO_2-to-SAF

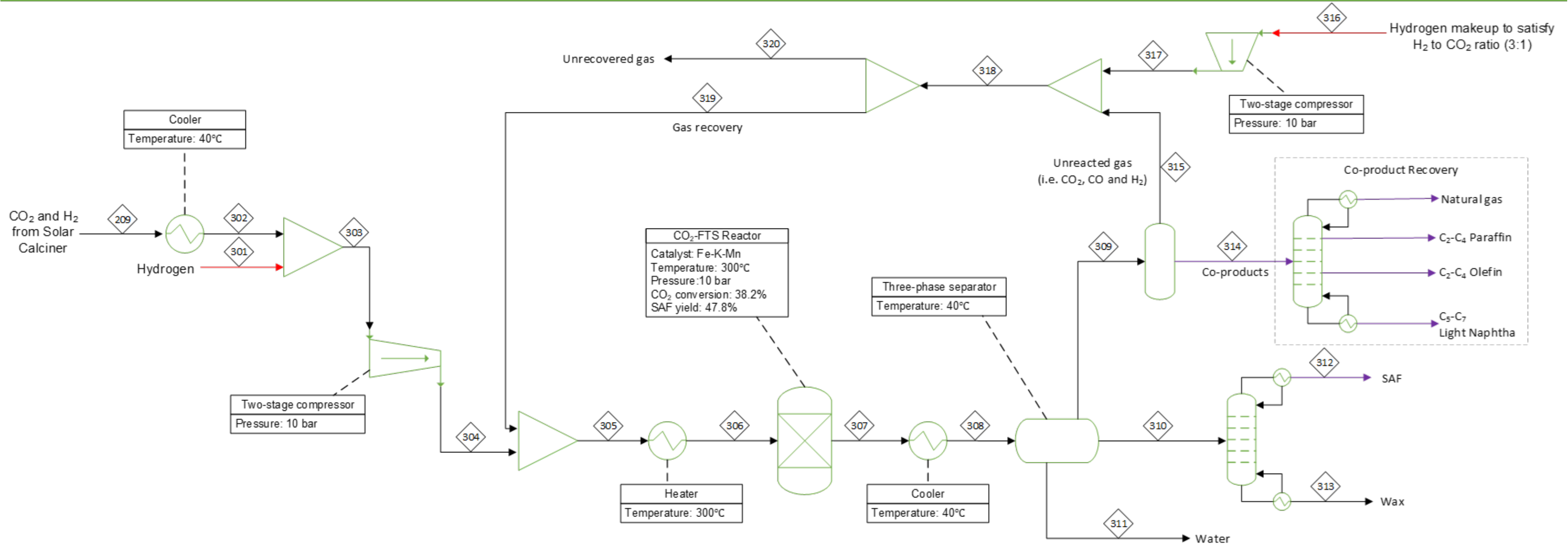

Component	Units	301	302	303	304	305	306	307	308	309	310	311	312	313	314	315	316	317	318	319	320
Total Flow	t/hr	21.3	407.4	407.4	407.4	929.4	929.4	929.4	929.4	632.7	51	245.7	46.2	4.8	55.8	577.2	2.7	2.7	579.9	521.7	57.9
Temperature	°C	21	40	33	40	40	300	300	40	40	40	40	40	40	40	40	21	40	40	40	40
Pressure	bar	1	1	1	10	10	10	10	10	10	10	10	10	10	10	10	1	10	10	10	10
CO	t/hr					10.41	10.41	11.55	11.55	11.55	0	0		0		11.55			11.55	10.41	1.17
H_2	t/hr	21.21	28.02	49.23	49.23	113.01	113.01	68.1	68.1	68.1	0	0		0		68.1	2.73	2.73	70.86	63.75	7.08
CO_2	t/hr		358.26	358.26	358.26	805.95	805.95	498.09	498.09	497.43	0.63	0.03		0.63		497.43			497.43	447.69	49.74
H_2O	t/hr							251.31	251.31	5.61	0.12	245.55		0.12	5.61						
CH_4	t/hr							11.31	11.31	11.31	0	0		0	11.31						
C_2H_6	t/hr							4.23	4.23	4.23	0	0		0	4.23						
C_2H_4	t/hr							5.22	5.22	5.22	0	0		0	5.22						
C_3H_8	t/hr							4.89	4.89	4.86	0.03	0		0.03	4.86						
C_3H_6	t/hr							7.23	7.23	7.2	0.03	0		0.03	7.2						
C_4H_{10}	t/hr							3.18	3.18	3.12	0.06	0		0.06	3.12						
C_4H_8	t/hr							2.79	2.79	2.73	0.06	0		0.06	2.73						
C_5H_{12}	t/hr							2.82	2.82	2.67	0.15	0		0.15	2.67						
C_5H_{10}	t/hr							2.1	2.1	2.01	0.09	0		0.09	2.01						
C_6H_{14}	t/hr							2.82	2.82	2.19	0.27	0		0.27	2.19						
C_6H_{12}	t/hr							2.1	2.1	1.44	0.24	0		0.24	1.44						
C_7H_{16}	t/hr							2.19	2.19	1.5	0.69	0		0.69	1.5						
C_7H_{14}	t/hr							1.32	1.32	0.9	0.42	0		0.42	0.9						
$C_{12}H_{26}$	t/hr							43.08	43.08	0.63	42.45	0	42.45	0	0.63						
$C_{12}H_{24}$	t/hr							3.9	3.9	0.06	3.84	0	3.84	0	0.06						
$C_{20}H_{42}$	t/hr							1.89	1.89	0	1.89	0		1.89	0						

Supplementary Figure 4: Process flow information of CO_2-to-SAF section.

Supplementary Note 2: Simulation and comparison of DAC process at commercial scale

The chemistry of CO_2 absorption with aqueous KOH is described by the set of equilibrium reactions (Supplementary Table 1) and the kinetic reactions (Supplementary Table 2).

Supplementary Table 1: Chemistry settings of equilibrium reactions for rate-based air contactor model.

$lnK = A + B/T + ClnT + DT$				
Equilibrium Reactions	A	B	C	D
$\mathrm{CO_2 + 2H_2O \leftrightarrow HCO_3^- + H_3O^+}$	231.465	-12092.1	-36.7816	0
$\mathrm{HCO_3^- + H_2O \leftrightarrow H_3O^+ + CO_3^{2-}}$	216.05	-12431.7	-35.4819	0
$\mathrm{2H_2O \leftrightarrow OH^- + H_3O^+}$	132.899	-13445.9	-22.4773	0

Supplementary Table 2: Chemistry settings of kinetic reactions for rate-based air contactor model.

$Kinetic\ factor = KT^n e^{-\frac{E}{RT}}$			
Kinetic Reactions	K	n	E (J/kmol)
$\mathrm{CO_2 + OH^- \rightarrow HCO_3^-}$	4.32e+13	0	5.54709e+07
$\mathrm{HCO_3^- \rightarrow CO_2 + OH^-}$	2.38e+17	0	1.23305e+08

The simulation approach of one modular unit of air contactor was adopted from Sabatino et al.[1]. This model effectively represents the air contactor unit design presented by Keith et al.[2]. Furthermore, we enhanced the rate-based model by incorporating modified built-in packing allowing us to represent the Brentwood XF12560 packing accurately, and to correlate the packing pressure drop based on pilot experiments.

Since the Brentwood XF12560 packing is not a built-in option in Aspen Plus®, we used a modified Sulzer 250Y PVC packing to represent it. The specific information regarding the Brentwood XF12560 packing—such as the specific surface area (SSA), void fraction and liquid holdup—is provided in the table below and was used to modify the default data for the Sulzer 250Y PVC packing.

Supplementary Table 3: Modified Sulzer 250Y PVC packing setting in Aspen Plus®.

Parameter	Settings	Reference
Material	PVC	Holmes (2010)[3]
SSA	210 m^2/m^3	
Liquid hold up	35 g/m^2	
Void fraction	0.95	Cooling Tower Depot® (2023)[4] and Fair et al. (2000)[5]

Calculation of packing pressure drop:

The pressure drop was estimated using the correlations developed for Brentwood XF12560 packing. This pressure drop correlation provided by Brentwood Industries Inc.[6] was adapted from the pilot experimental data (air velocity between 1-2 m/s) given in Keith et al.[2].

$$\Delta P = \left(7.4 {v_{air}}^{2.14a} + 4.3 Q_l \times {v_{air}}^{0.4b}\right) \times ATD^{0.95c}$$

Where ΔP is the pressure drop in Brentwood XF12560 packing (Pa), v_{air} is the air velocity driven by the air contactor (m/s), Q_l is the average liquid flow rate on the unit packing area (L/m^2 s), ATD is the air travel distance (m), a, b, c are fitting parameters (a=1.06, b=3.31, c=0.79).

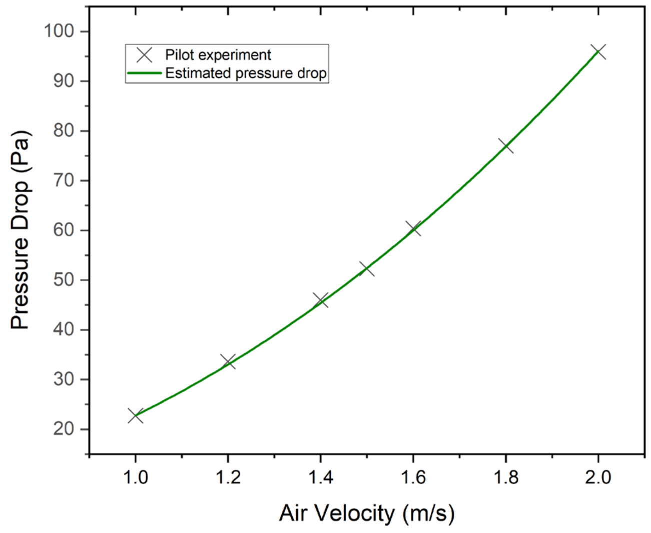

Supplementary Figure 5: Validation of Brentwood XF12560 packing pressure drop curve under 3 m ATD.

Calculation of fan electricity:

Electricity consumption for the fan is derived from Holmes and Keith[7]:

$$E_{fan} = 0.001\frac{\mathrm{A} \times \Delta P \times v_{air}}{\eta_{fan}}$$

Where, E_{fan} is the electricity consumption of the fan (kW). A is the total cross-sectional area of the air contactor (m^2) and the value is 40,000 m^2 for a commercial-scale air contactor. η_{fan} is the fan energy efficiency (%) and 70% fan efficiency is assumed[2].

Calculation of pumping electricity:

The pumping electricity is determined by loading rate, fluidisation velocity and pumping efficiency. Here, we assumed the same fluidisation velocity (0.6 L/m^2s) and the same pumping efficiency (82%) from Carbon Engineering's DAC design[2]. Therefore, the difference in pumping electricity consumption is calculated based on different loading rates according to mechanical energy requirements for the pumps[8].

Supplementary Table 4: Specifications of unit operations.

Parameter	Value	Source
**Air Contactor**		
Number of banks	10	Holmes et al. (2013)[9]
Number of air contactor units in each bank	160	
Number of blocks in each air contactor unit	6	Sabatino et al. (2021)[1]
Number of stages per block	20	
Diameter	5.64 m	
Height	1.16 m	
Packing	Modified Sulzer 250Y PVC (representing Brentwood XF12560 packing)	Holmes (2010)[3]
Solvent recovery rate	99.93%	Madhu et al. (2021)[10]
Operating temperature	21 ℃	Sabatino et al. (2021)[1]
Operating pressure	1 bar	
**Pellet Reactor**		
Operating temperature	21 ℃	Sabatino et al. (2021)[1]
Operating pressure	1 bar	
Calcium retention	90%	Keith et al. (2018)[2]
**Steam Slaker**		
Pellet water carryover	11.7 wt%	Adapted from process mass balance
Operating temperature	300 ℃	Keith et al. (2018)[2]
Operating pressure	1 bar	
CaO conversion	85%	
**Calciner**		
Operating temperature	900 ℃	Keith et al. (2018)[2]
Operating pressure	1 bar	
$CaCO_3$ conversion	98%	
**CO_2 Absorber**		
Diameter	7.5 m	Keith et al. (2018)[2]
Height	12 m	
Packing	BERL Ceramic packing	

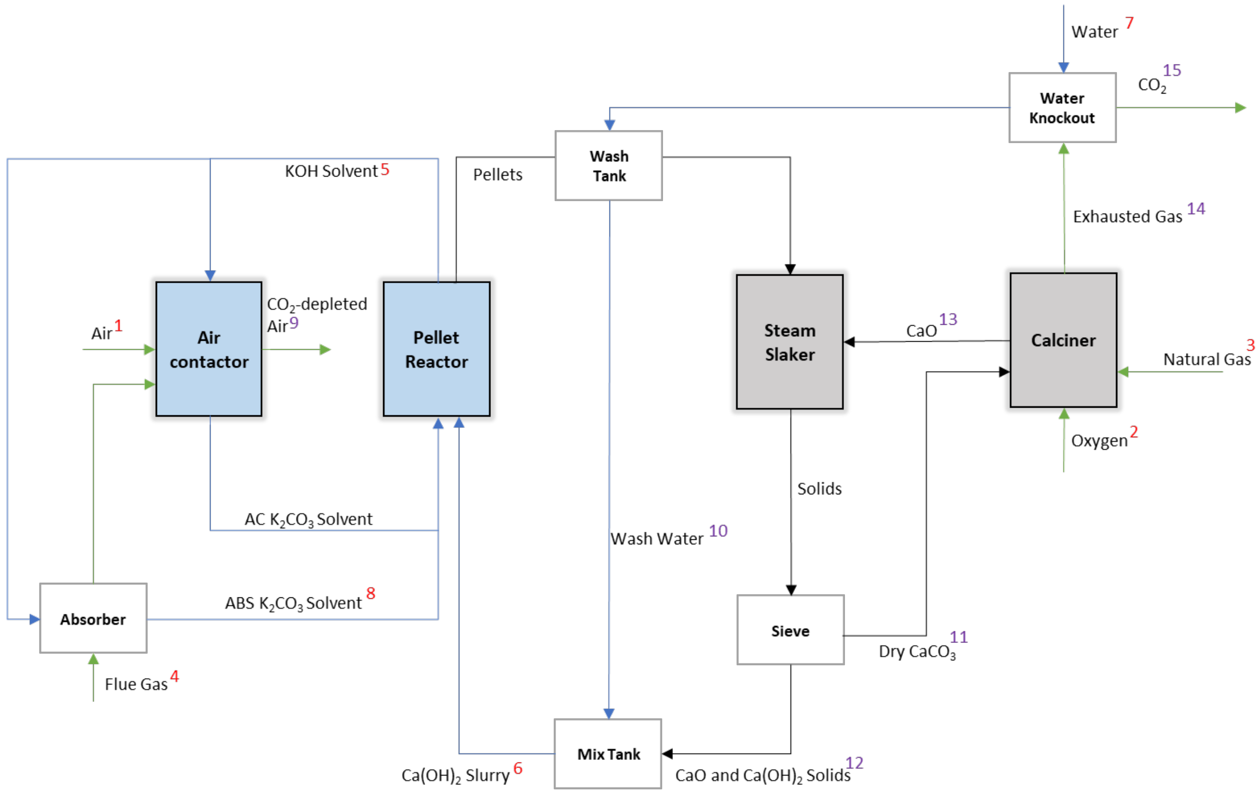

Supplementary Figure 6: Block flow diagram of Carbon Engineering's DAC process developed by Keith et al. to which our simulation is compared. The green arrow line lines represent gaseous streams, the blue arrow lines represent liquid streams, and the black arrow lines represent solid streams. The streams numbered 1-8 in red are the input streams, while the streams numbered 9-15 in purple are the outputs from the simulation to which our results are compared.

Supplementary Table 5: Inputs into the DAC process model.

Stream Number	Stream ID	Parameters	Value
1	Air	Temperature (°C)	21
		Flow rate (kt/hr)	251
		CO_2 (wt%)	0.06
		O_2 (wt%)	23.00
		N_2 (wt%)	75.96
		H_2O (wt%)	0.98
2	Oxygen	Temperature (°C)	21
		Flow rate (t/hr)	58.5
		O_2 (wt%)	95.60
		N_2 (wt%)	4.4
3	Natural Gas	Temperature (°C)	21
		Flow rate (kt/hr)	13.4
4	Flue Gas	Temperature (°C)	40
		Flow rate (kt/hr)	121
		CO_2 (wt%)	14.43
		O_2 (wt%)	0.91
		N_2 (wt%)	72
		H_2O (wt%)	12.66
5	KOH Solvent	Temperature (°C)	21
		Flow rate (kt/hr)	35
		K^+ (mol/L)	2
		OH^- (mol/L)	1.1
		CO_3^{2-} (mol/L)	0.45
6	$Ca(OH)_2$ Slurry	Temperature (°C)	21
		Flow rate (t/hr)	777
		$Ca(OH)_2$ (wt%)	28.7
7	Water	Temperature (°C)	21
		Flow rate (t/hr)	531
8	ABS K_2CO_3 Solvent	Temperature (°C)	31
		Flow rate (t/hr)	3200
		K^+ (mol/L)	2.01
		OH^- (mol/L)	0.68
		CO_3^{2-} (mol/L)	0.66

Supplementary Table 6: Model stream results and comparison with stream results from the Carbon Engineering's DAC process.

Stream ID	Name	Parameters	Carbon Engineering's DAC process	Simulation results	Relative error (%)
9	CO_2-depleted Air	Temperature (°C)	19	18.47	-2.79
		Flow rate (kt/hr)	252	251.55	-0.18
		CO_2 (wt%)	0.016	0.01542	-3.63
		O_2 (wt%)	22.96	22.95	-0.04
		N_2 (wt%)	75.83	75.8295	0
		H_2O (wt%)	1.2	1.2	0
10	Wash Water	Temperature (°C)	NA	54	NA
		Flow rate (t/hr)	567	566.998	0
		K^+ (kg/hr)	3089	3080	-0.29
		OH^- (kg/hr)	768	740	-3.65
		CO_3^{2-} (kg/hr)	1016	1062	4.53
11	Dry $CaCO_3$	Temperature (°C)	300	300	0
		Flow rate (t/hr)	300	300.58	0.19
12	CaO and $Ca(OH)_2$ Solids	Temperature (°C)	300	300	0
		Flow rate (t/hr)	211	210.11	-0.42
		CaO (t/hr)	24.7	24.76	0.24
		$Ca(OH)_2$ (t/hr)	186	185.35	-0.35
13	CaO	Temperature (°C)	674	674	0
		Flow rate (t/hr)	165	165.04	0.02
14	Exhausted Gas	Temperature (°C)	454	450	-0.88
		Flow rate (t/hr)	201	201.41	0.20
		CO_2 (wt%)	82.57	82.553	-0.02
		O_2 (wt%)	1.16	1.227	5.78
		N_2 (wt%)	1.28	1.278	-0.16
		H_2O (wt%)	14.99	14.942	-0.32
15	CO_2	Flow rate (t/hr)	171	171.33	0.19
		CO_2 (wt%)	97.12	97.046	-0.08
		O_2 (wt%)	1.36	1.442	6.03
		N_2 (wt%)	1.51	1.502	-0.53
		H_2O (wt%)	0.01	0.0098	-2.00

Supplementary Table 7: Model unit performance and comparison with unit performance from Carbon Engineering's DAC process.

Parameter	Carbon Engineering's DAC process	Simulation results	Relative error (%)
**Air Contactor**			
CO_2 captured level (%)	74.5	74.54	0.05
Pumping energy (kWh/t CO_2)	21	20.9	0.13
Fan energy (kWh/t CO_2)	61	61.2	0.56
**Pellet Reactor**			
Ca^{2+} to $CaCO_3$ conversion (%)	100[d]	100	0
Discharged pellets (%)	~90	90	0
Pumping energy (kWh/t CO_2)	27	26.9	0.14
**Steam Slaker**			
CaO conversion (%)	85	85	0
Slaking heat (kJ/mol)	105.2[11]	105.03	-0.16
**Calciner**			
$CaCO_3$ conversion (%)	98	98	0
Thermal energy consumption (GJ/t CO_2)	5.25	5.23	-0.44
**CO_2 absorber**			
CO_2 absorber capture level (%)	90	89.99	-0.01

Supplementary Note 3: Modelling, simulation and validation of solar calciner at pilot scale.

This pilot-scale solar reactor is a four-stage fluidised bed fluidised by air from the bottom and particles cross-flow from the first stage to the fourth stage. The solar reactor is 1 m long and contains 4 compartments each 25 cm long, 8 cm wide and separated from each other by 40 cm high baffles. The solar reactor can process 40 kg particles at 0.5 void fraction. The experiments showed the highest conversion at 95.2% which was conducted at 20 kg/h particles and a high air fluidisation flow rate of 30 Nm^3/h. In this case, the highest thermochemical efficiency (17%) and highest thermal efficiency (29%) were achieved.

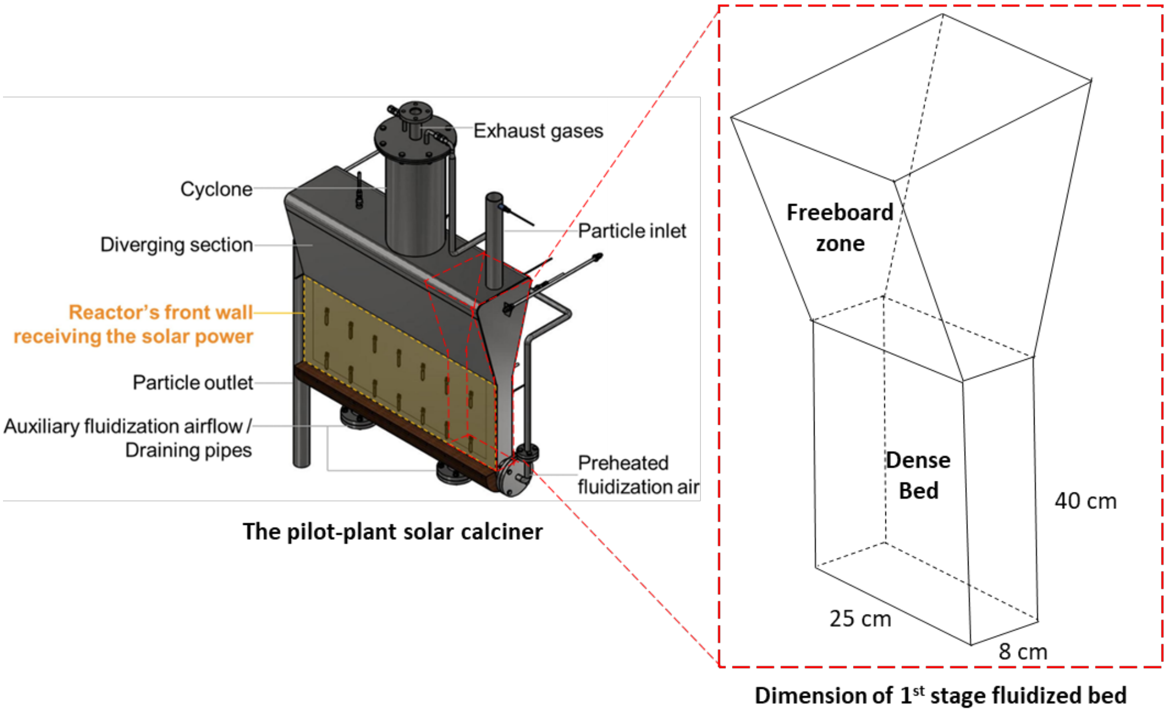

The pilot-plant solar calciner

Dimension of 1st stage fluidized bed

Supplementary Figure 7: Schematic diagram of the pilot-scale solar reactor and the dimension of 1st stage fluidised bed.

Assumptions

- The solar calciner is modelled at steady-state.
- The operating temperature represents the mean reactor temperature.
- The hydrodynamics of the fluidised bed are only considered on the height axis.

Modelling and simulation

To reproduce the four-stage horizontal fluidised bed used for solar calcination, the solar reactor was modelled and simulated in four fluidised beds arranged in series, as represented in the following Figure. The process flowsheet of this new modelling design was conducted in Aspen Plus® V11. Aspen Custom Modeller® (ACM) V11 acted as an additional calculation block in this flowsheet to correct the steam enthalpy and calculate the thermal efficiency.

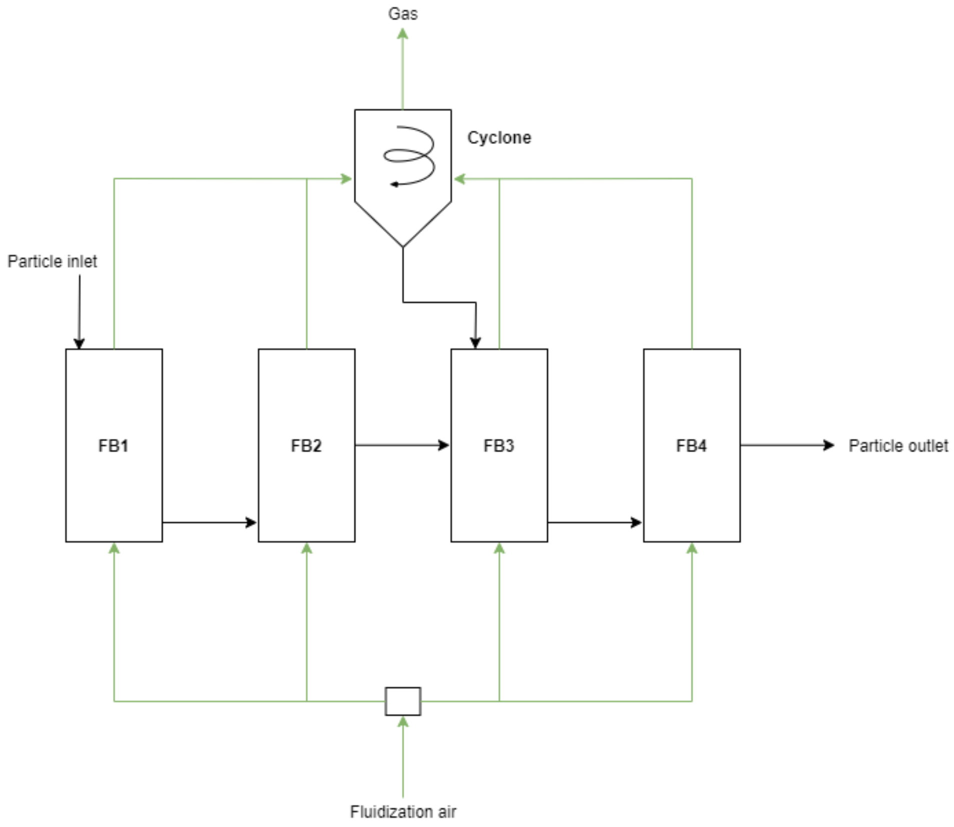

Supplementary Figure 8: Representation of the approach adopted to model the four-stage horizontal solar fluidised bed (FB: fluidised bed).

Supplementary Table 8: Specification of fluidised bed

**Bed geometry**	
Bed number	4
Width	25 cm
Depth	8 cm
Height (dense bed height)	40 cm
1st stage solid discharge location	5 cm
2nd stage solid discharge location	30 cm
3rd stage solid discharge location	5 cm
4th stage solid discharge location	40 cm
**Bed inventory**	
Voidage at minimum fluidisation	0.5
Geldart classification	Geldart A
**Other model settings**	
Minimum fluidisation velocity correlation	Wen & Yu (1966)
Transport disengagement height	Fung and Hamdullahpur (1993)
Elutriation	Tasirin & Geldart (1998)

Supplementary Table 9: Enthalpy correlation and thermal efficiency calculation

Parameter	Reference
Heat of reaction	Robie and Hemingway, 1995[12]
Enthalpy of $CaCO_3$	Robie and Hemingway, 1995[12]
Enthalpy of Air	Hilsenrath et al., 1955[13]
Thermochemical efficiency	Esence et al., 2020[14]
Thermal efficiency	Esence et al., 2020[14]

Supplementary Table 10: Process specifications of solar calcination process at pilot scale

Parameter	Case 1: 20 kg/hr low fluidisation flow rate	Case 2: 20 kg/hr high fluidisation flow rate
Mass flow rate of $CaCO_3$ (kg/hr)	20	20
Air flow rate (Nm^3/hr)	10.1	19.3
Auxiliary air (Nm^3/hr)	3	4
Global temperature in calciner (℃)	831	813
Solar power in (kW)	67.4	57.8
Inlet $CaCO_3$ purity (wt%)	98.5%	98.5%

Supplementary Table 11: Validation of two representative experimental data of solar calcination

	Case 1: 20 kg/hr low fluidisation flow rate			Case 2: 20 kg/hr high fluidisation flow rate		
	**Experimental data**	**Simulation result**	**Relative Errors**	**Experimental data**	**Simulation result**	**Relative Errors**
**Mass balance**						
$CaCO_3$ conversion	88.50%	88.57%	-0.08%	95.20%	95.43%	-0.24%
$CaCO_3$ weight fraction in solids outlet	18.60%	18.26%	1.80%	7.62%	7.68%	-0.75%
CaO weight fraction in solids outlet	80.65%	80.92%	-0.34%	91.24%	91.22%	0.03%
Unreactive materials weight fraction in solids outlet	0.75%	0.81%	-8.16%	1.14%	1.11%	2.81%
**Energy balance**						
Thermochemical efficiency	14%	13.62%	2.70%	17%	17.10%	-0.60%
Thermal efficiency	23%	23.42%	-1.85%	29%	29.13%	-0.46%

Supplementary Note 4: Modelling, simulation and validation of CO_2-to-SAF at lab scale

Assumptions

The following assumptions were made for the CO_2 utilisation model using the CO_2-FTS approach.

- This model, which transforms CO_2 into jet fuel, operates at steady-state. Hence, there is no accumulation of heat and mass in the fixed bed reactor.
- Only reaction conversion and specific products are considered based on material balance and stoichiometric reactions.
- Only olefin and paraffin are considered for hydrocarbons in this model (depending on the catalyst nature, the selectivity of oxygenates was neglected in experiments due to it being below 1.0%), and the ratio of olefin to paraffin for each hydrocarbon is obtained from jet fuel experiments[15,16].
- The chain growth probability of hydrocarbons is based on the given Anderson-Schulz-Flory (ASF) plot from experiments[15].
- The lumping technique was used for a large number of products from CO_2-FTS reactions. The infinite number of hydrocarbon components is handled by the lump of components from C_1 to C_{50}.

Modelling and simulation of the CO_2-FTS process

A modified ASF model combining ASF theory and kinetic modelling on the Fischer-Tropsch reaction was implemented in ACM to predict the hydrocarbon distribution of converting CO to jet fuel under Fe-Mn-K catalyst in the carbon number range from C_1 to C_{50}.

C_3 compounds do not follow the theoretical ASF distribution and are removed from the modified ASF model. The chain growth probability is calculated separately based on the kinetic values proposed by Kamkeng and Wang[17].

Based on the predicted selectivity of products in ACM, the mass and energy balance of the CO_2-FTS process using the lumping technique was simulated in Aspen Plus® V11.

Supplementary Table 12: Equations of CO_2-FTS model implemented in ACM.

Parameter	Equation	Reference
Chain growth probability of C3 (α_{C_3})	$\alpha_{C_3} = \frac{k_1 P_{CO_2}}{k_1 P_{CO_2} + k_5 P_{H_2} + k_{6,0} e^{3c}}$	Kamkeng and Wang (2023)[17], Todic et al. (2013)[18]
Weight fraction of C3 (W_3)	$W_3 = 3 \times (1 - \alpha_{C_3})^2 \times {\alpha_{C_3}}^2$	
Product fraction related to α_1 (f_1)	$f_1 = \lambda \frac{1 - W_3}{\frac{1}{1-\alpha_1} - \frac{\alpha_1}{1+\alpha_1} + \left[\frac{1}{1-\alpha_2} - \frac{\alpha_2}{1+\alpha_2}\right](\frac{\alpha_1}{\alpha_2})^{b-1}}$	Kamkeng and Wang (2023)[17]
Product fraction related to α_2 (f_2)	$f_2 = f_1 \times (\frac{\alpha_1}{\alpha_2})^{b-1}$	
Weight fraction of hydrocarbon n (W_n)	$W_n = f_1 \times {\alpha_1}^{n-1} + f_2 \times {\alpha_2}^{n-1}$	Donnelly et al. (1988)[19]
Average molecular weight of hydrocarbons ($\bar{M}$)	$\frac{1}{\bar{M}} = \sum_{n=1}^{50} \frac{W_n}{M_n}$	Kamkeng and Wang (2023)[17]
Mole fraction of hydrocarbon n (x_n)	$x_n = \frac{W_n}{M_n} \times \bar{M}$	
Selectivity of hydrocarbon n (S_n)	$S_n = \frac{x_n \times n}{\sum_{n=1}^{50} x_n \times n}$	
Total molar flow rate of hydrocarbons (N_{HC})	$N_{HC} = n_{CO_{2in}} \times X_{CO_2} \times X_{CO}$	
Molar flow rate of hydrocarbon n (n_{HC})	$n_{HC} = N_{HC} \times \frac{S_n}{n}$	

Supplementary Table 13: Products and chemical reactions implemented for the CO_2-FTS model.

Carbon range	Product category	Carbon number	Component	Chemical reaction
n=1	Carbon Monoxide	1	CO	$CO_2 + H_2 \rightarrow CO + H_2O$
	Methane		CH_4	$CO + 3H_2 \rightarrow CH_4 + H_2O$
$2 \leq n \leq 4$	Light Hydrocarbon (C_2-C_4)	2, 3 and 4	C_2H_6	$2CO + 5H_2 \rightarrow C_2H_6 + 2H_2O$
			C_2H_4	$2CO + 4H_2 \rightarrow C_2H_4 + 2H_2O$
			C_3H_8	$3CO + 7H_2 \rightarrow C_3H_8 + 3H_2O$
			C_3H_6	$3CO + 6H_2 \rightarrow C_3H_6 + 3H_2O$
			C_4H_{10}	$4CO + 9H_2 \rightarrow C_4H_{10} + 4H_2O$
			C_4H_8	$4CO + 8H_2 \rightarrow C_4H_8 + 4H_2O$
$5 \leq n \leq 7$	Light Naphtha (C_5-C_7)	5, 6 and 7	C_5H_{12}	$5CO + 11H_2 \rightarrow C_5H_{12} + 5H_2O$
			C_5H_{10}	$5CO + 10H_2 \rightarrow C_5H_{10} + 5H_2O$
			C_6H_{14}	$6CO + 13H_2 \rightarrow C_6H_{14} + 6H_2O$
			C_6H_{12}	$6CO + 12H_2 \rightarrow C_6H_{12} + 6H_2O$
			C_7H_{16}	$7CO + 15H_2 \rightarrow C_7H_{16} + 7H_2O$
			C_7H_{14}	$7CO + 14H_2 \rightarrow C_7H_{14} + 7H_2O$
$8 \leq n \leq 16$	Jet Fuel (C_8-C_{16})	12	$C_{12}H_{26}$	$12CO + 25H_2 \rightarrow C_{12}H_{26} + 12H_2O$
			$C_{12}H_{24}$	$12CO + 24H_2 \rightarrow C_{12}H_{24} + 12H_2O$
$n \geq 17$	Wax (C_{17+})	20	$C_{20}H_{42}$	$20CO + 41H_2 \rightarrow C_{20}H_{42} + 20H_2O$

Supplementary Table 14: Input parameters for CO_2-FTS model validation

<table>
<tr><th colspan="2">Parameter</th><th>Value</th><th>Reference</th></tr>
<tr><td colspan="2">Reactor type</td><td>fixed bed</td><td rowspan="7">Yao et al. (2020)[15]</td></tr>
<tr><td colspan="2">Reactor diameter (cm)</td><td>1</td></tr>
<tr><td colspan="2">Reactor temperature (℃)</td><td>300</td></tr>
<tr><td colspan="2">Reactor pressure (MPa)</td><td>1</td></tr>
<tr><td colspan="2">H_2/CO_2 ratio</td><td>3</td></tr>
<tr><td colspan="2">Flow rate (mL/min)</td><td>40</td></tr>
<tr><td colspan="2">CO_2 conversion (%)</td><td>38.2</td></tr>
<tr><td rowspan="2">Chain growth probability</td><td>α_1</td><td>0.79</td><td rowspan="3">Donnelly et al. (1988)[19], Yao et al. (2020)[15]</td></tr>
<tr><td>α_2</td><td>0.572</td></tr>
<tr><td colspan="2">Carbon number at breakpoint</td><td>12</td></tr>
<tr><td rowspan="3">Kinetic constants</td><td>k_1</td><td>1.66×10^{-2}</td><td rowspan="7">Kamkeng and Wang (2023)[17], Todic et al. (2013)[18]</td></tr>
<tr><td>k_5</td><td>6.99×10^{-5}</td></tr>
<tr><td>$K_{6,0}$</td><td>2.02×10^{-2}</td></tr>
<tr><td colspan="2">Constant c</td><td>-0.26</td></tr>
<tr><td rowspan="3">Fitting parameter λ</td><td>$n \leq 7$</td><td>$0.15e^{0.35n}$</td></tr>
<tr><td>$8 \leq n \leq 12$</td><td>$0.29e^{0.39n}$</td></tr>
<tr><td>$n \geq 13$</td><td>$2.7e^{36n^{-1}}$</td></tr>
</table>

Supplementary Table 15: Model validation results of selectivity for different product categories

**Product category**	**Selectivity (%)**		**Relative error (%)**
	Experimental data	Model prediction	
**CO**	5.6	5.60	0.00
**C_1**	10.4	10.14	-2.51
**C_2-C_4**	27.7	27.60	-0.34
**C_5^+**	61.9	62.26	0.58
**C_8-C_{16}**	47.8	47.64	-0.34

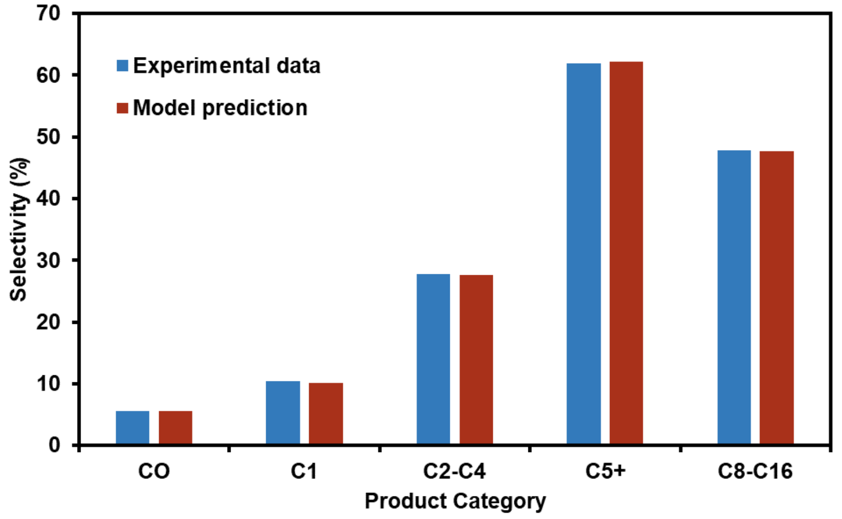

Supplementary Figure 9: Model predictions and experimental values of CO_2-FTS product selectivity.

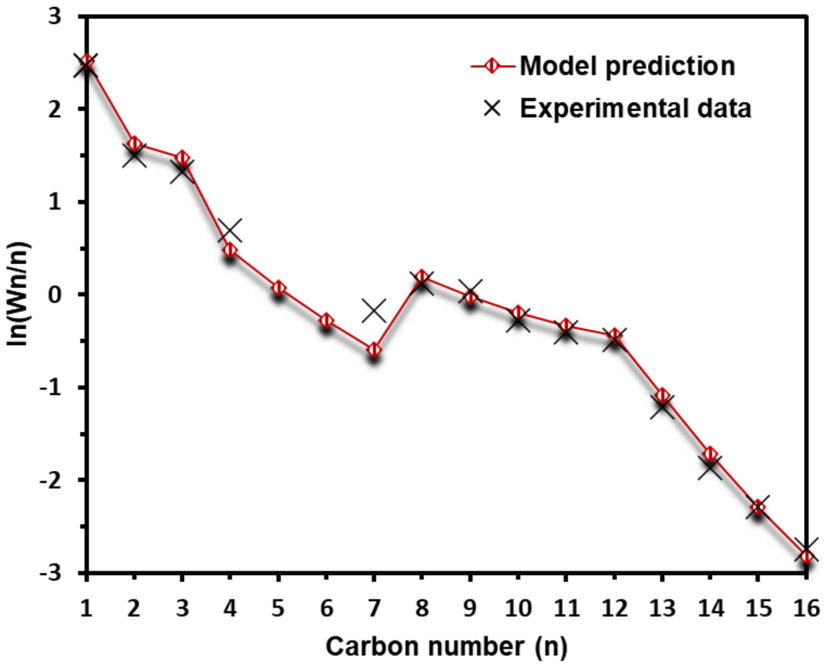

Supplementary Figure 10: Anderson-Schulz-Flory plots showing the prediction of hydrocarbon distributions.

Supplementary Note 5: Scale-up approach of fluidised bed

Kelkar and Ng studied the scale-up of fluidised beds with the consideration of both hydrodynamic similarity and reaction converion[20]. Based on the scaling law, the bubble size of the fluidised bed is controlled in a suitable range to achieve the same reaction conversion at a large scale. With the commercial-scale solar calciner, we can achieve the large-scale CSP-driven DAC. The reactor geometry and operation parameters will be based on operating and economic optimisation.

Hydrodynamics similarity (Horio et al., 1986)

$$n = \frac{D_T^l}{D_T^s} = \frac{H^l}{H^s} = \frac{P_n^l}{P_n^s} = \frac{d_b^l}{d_b^s}$$

$$n^{0.5} = \frac{U_{mf}^l}{U_{mf}^s} = \frac{U_G^l}{U_G^s} = \frac{u_b^l}{u_b^s}$$

$$1 = \frac{\epsilon_s^l}{\epsilon_s^s} = \frac{\epsilon_b^l}{\epsilon_b^s}$$

$$n^3 = \frac{F_s^l}{F_s^s}$$

$$n^{2.5} = \frac{F_G^l}{F_G^s}$$

Supplementary Figure 11: Scale-up principle of solar calciner for achieving hydrodynamic similarity.

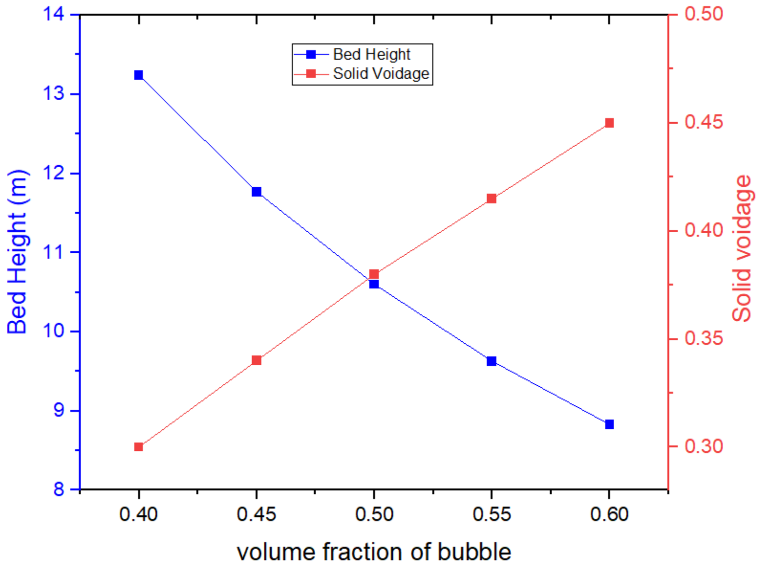

Supplementary Figure 12: Bed height and solid voidage as a function of bubble volume fraction at large-scale.

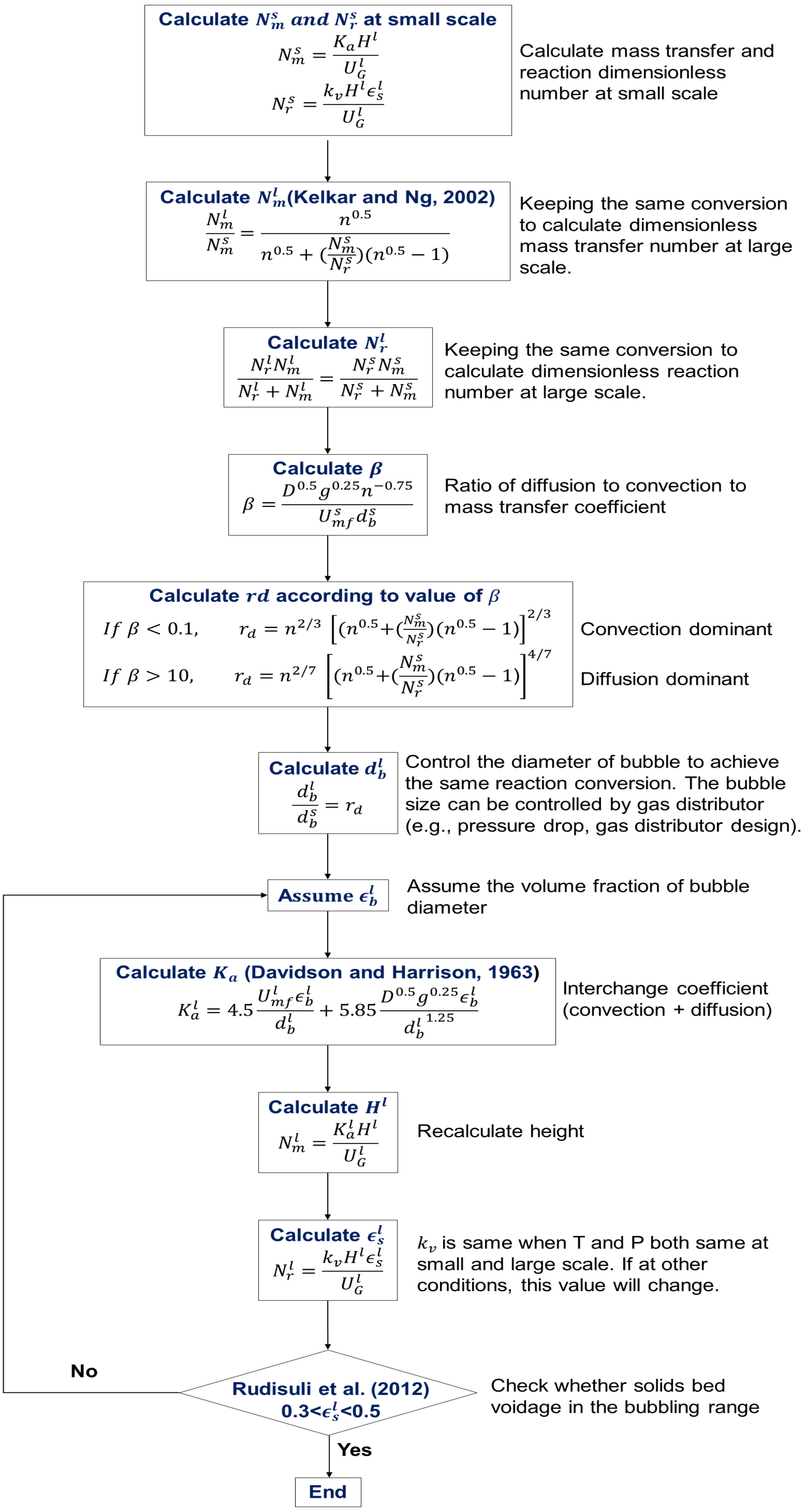

Supplementary Figure 13: Scale-up principle of solar calciner for achieving the same reaction conversion.

Supplementary Table 16: Example of large-scale hydrogen-based solar calciner through scaling approach.

Symbol	Description	Unit	Pilot-scale (Air)	Pilot-scale (H_2)	Commercial-scale (H_2)
n	scaling factor		1		10
			Reactor Geometry and Phase Flows		
L	Length	m	0.08	0.08	0.8
W	Width	m	0.25	0.25	2.5
H	Height	m	0.8	0.8	10.73
F_S	Solids flow rate	kg/h	20	20	20,000
F_G	Gas flow rate	kg/h	30	2.077	656.8
U_{mf}	Minimum fluidisation velocity	mm/s	4	8.1	25.5
U_G	Superficial fluidisation velocity	m/s	0.4	0.404	1.28
u_b	Bubble velocity	m/s	0.95	0.94	2.97
			Phase distribution		
d_p	Mean particle diameter	µm	100	100	178
d_b	Bubble diameter	m	0.053	0.05	0.7
ε_b	Volume fraction of bubble	-	0.415	0.42	0.5
ε_s	Voidage of solids	-	0.5	0.5	0.37
			Transport attributes		
K_a	Interchange coefficient	s^{-1}	0.64	0.01	0.69
			Dimensionless No. and Reactor Performance		
N_r	Reaction numbers	-	1.28	1.49	4.69
N_m	Mass transfer numbers	-	0.1	1.37	0.84
X_{CaCO3}	$CaCO_3$ conversion	-	0.95	0.95	0.95

Supplementary Note 6: Techno-economic assessment

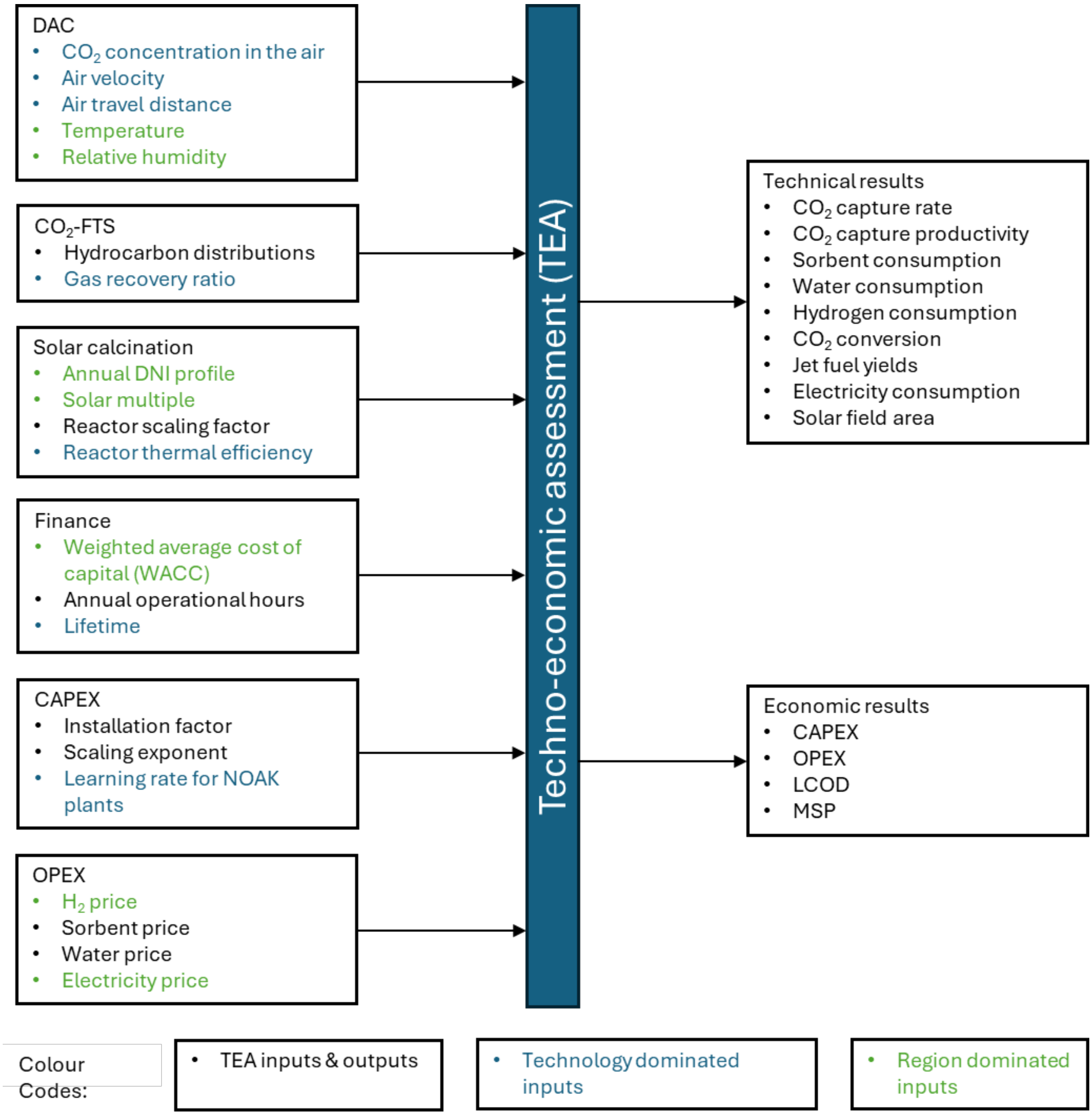

Supplementary Figure 14: Techno-economic assessment framework.

Supplementary Table 17: Equipment installed cost breakdown by section at base case.

Equipment type	Installation factor	Scaling exponent	Reference installed cost (US$)	Installed cost (2020 US$)	Source
**Direct air capture**					
Air contactor	1.86	1	212,200,000	233,549,271	[2]
Pellet reactor	1.7	0.675	130,700,000	133,967,959	[2]
Steam slaker	1.45	0.675	38,850,000	37,606,988	[2]
Steam turbine	NA	0.7	7,510,000	8,265,575	[2]
Filter	1.76	NA	30,900,000	31,840,017	[2]
Other equipment	NA	NA	102,900,000	89,045,962	[2,21]
Buildings	NA	0.61	6,700,000	6,566,310	[2]
Transformer	NA	0.7	19,800,000	19,075,577	[2]
Subtotal				559,917,659	
**Solar calcination**					
Heliostat field	NA	1	NA	126,688,860	[22]
Parabolic mirror	2.27	1	NA	38,006,658	[22]
Solar calciner & solar tower	2.27	0.48	NA	211,679,219	[21]
$CaCO_3$ storage tank	NA	NA	NA	1,556,530	[21]
CaO storage tank	NA	NA	NA	1,006,128	[21]
Subtotal				378,937,395	
**CO_2-to-SAF**					
Mixed gas cooler	3.23	0.7	4,186,906	6,414,481	[23]
Mixed gas compressor	2.47	0.65	11,461,600	56,825,619	[23]
H_2 compressor	2.47	0.6	2,547,995	1,108,176	[23]
Preheater	2.47	0.6	6,248,204	14,770,241	[23]
CO_2-FTS reactor	2.75	0.8	36,681,585	262,237,754	[23]
Syncrude cooler	1.52	0.7	50,244	1,455,142	[23]
Three-phase separator	1.69	0.7	6,595,713	17,995,594	[23]
Distillation unit	2.47	0.65	2,547,995	74,788,647	[23,24]
Subtotal				435,596,192	

Equipment of DAC is quoted from CE's DAC design and updated based on size and cost to 2020. Other equipment is assumed to be 20% of the cost for the rest of the process equipment, including cyclones, mix tank and auxiliary equipment. Heilostat field cost is according to US$75/$m^2$.Parabolic mirror cost is assumed as 30% of the heliostat field cost. Solar receiver and tower were calculated together. Cost of storage tanks were calculated according to the reference method[21]. Distillation unit is assumed as a holistic cost including jet fuel distillation, separator and co-product recovery system (distillation columns, compressors, heaters and coolers).

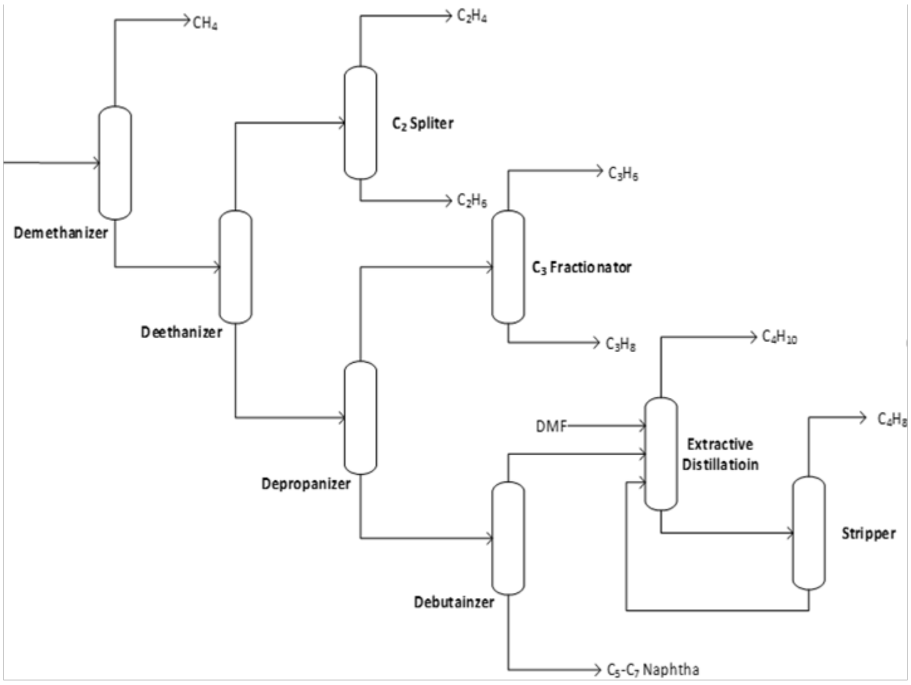

Equipment	Equipment Cost (2020)	Installed Cost (2020)	Installation factor	Saling exponent	Ref Equipment cost	Ref Installed cost	Model Capacity	Ref Capacity	Unit	Bace year	Ref
Demethanizer	1,456,373	2,907,122	2.00	0.65	**259,000**	**517,000**	50	4.06	t/h	2016	Yadav et al. (2021)
Deethanizer	1,176,874	2,010,493	1.71	0.65	240,000	410,000	38.7	3.88	t/h	2016	Yadav et al. (2021)
C2 splitter	1,506,794	2,495,627	1.66	0.65	256,000	424,000	9.40	0.71	t/h	2016	Yadav et al. (2021)
Depropanizer	756,281	1,414,525	1.87	0.65	162,000	303,000	29.3	3.17	t/h	2016	Yadav et al. (2021)
C3 Fractionator	4,253,699	5,526,370	1.30	0.65	1,113,000	1,446,000	12.1	1.78	t/h	2016	Yadav et al. (2021)
Debutanizer	423,637	1,112,754	2.63	0.65	75,000	197,000	17.2	1.39	t/h	2016	Yadav et al. (2021)
Extractive distillation	1,158,607	1,378,886	1.19	0.65	405,000	482,000	5.8	1.33	t/h	2016	Yadav et al. (2021)
Stripper	260,619	353,698	1.36	0.65	126,000	171,000	2.7	1.02	t/h	2016	Yadav et al. (2021)
**Subtotal**		$ 17,199,475									
**Total installed cost of co-product process (major equipment and other equipment) = $17,199,475 * 2.58 =$44,322,392**											

Supplementary Figure 15: Design and cost of co-product recovery system.

While the co-product recovery is illustrated as a single block in Supplementary Figure 4, for clarity, this unit consists of multiple components, including distillation columns, compressors, heaters and coolers. Specifically, the process handles a stream of 55.8 t/h, which contains 10.1 wt% water, 69.3 wt% C_1-C_4 hydrocarbons and 20.4 wt% C_5^+ hydrocarbons. After water removal, the remaining hydrocarbons can be separated by conventional petroleum refinery processes—a mature and well-established industrial process. Here, the same separation process was adopted from previous study[24] to recover high-purity (>99.5 wt%) fractions of CH_4, C_2H_4, C_2H_6, C_3H_6, C_3H_8, C_4H_8, C_4H_{10} and C_5-C_7 naphtha (see Supplementary Figure 15). The total installed cost of the co-product recovery system is $44.3 million, which is around 59.3% of the distillation unit cost.

Supplementary Table 18: CAPEX of 1 Mt_{CO2}/yr solar-driven DACCU plant by section at base case.

Parameter	Item	Calculation method	Cost (2020 US$M)
**Direct air capture**			
Total direct field costs (TDFC)		Sum of the installed cost	559.9
Indirect field costs (IFC)		12.7% of TDFC	71.1
Total field costs (TFC)		TDFC + IFC	631.0
Non-field costs (NFC)	Engineering	12% of TPC	108.4
	Contingency	6% of TFC	126.2
	Other project costs	20% of TFC	37.9
Total non-field costs (TNFC)		Sum of non-field costs	272.5
Total project costs (TPC)		TFC + TNFC	903.5
**Solar calcination**			
TDFC		Sum of the installed cost	378.9
Contingency		10% TDFC	37.9
Direct cost		TDFC + Contingency	416.8
Indirect cost		9% TDFC	34.1
Total capital investment of CSP		Direct cost + Indirect cost	450.9
**CO_2-to-SAF**			
TDFC		Sum of the installed cost	435.6
IFC		12.7% of TDFC	55.3
TFC		TDFC + IFC	490.9
NFC	Engineering	12% of TPC	84.3
	Contingency	6% of TFC	98.2
	Other project costs	20% of TFC	29.5
TNFC		Sum of non-field costs	212.0
TPC		TFC + TNFC	702.9
Land cost		Average land cost	21.1
Total CAPEX			2,078.5

Supplementary Table 19: Annual operational expenditure breakdown at base case.

Operating Parameters		
**Parameter**	**Value**	**Units**
Annual operating hours	8,000	hr/yr
CO_2 concentration in the air	420	ppm
Air velocity	1.4	m/s
Air travel distance	7	m
CO_2 capture rate	74.5	%
Annual CO_2 capture productivity	0.96	Mt/yr
Annual SAF productivity	0.12	Mt/yr
**Variable Operating Costs**		
**Raw material/Utility**	**Mass flow (kg/hr) or Energy flow (kW)**	**Cost (US$/yr)**
KOH makeup	907.7	5,446,008.0
$CaCO_3$ makeup	2,717.0	4,347,200.0
Water makeup	516,110.0	4,128,880.0
Fan electricity	6,858.8	1,646,112.0
Pumping electricity	5,028.8	1,206,912.0
Hydrogen for fluidisation	8,110.0	141,438,616.3
Additional hydrogen	8,300.5	144,760,503.7
Hydrogen makeup	912.7	15,917,413.0
Catalyst consumption	assumed as 2 t/yr[25]	7,960,000.0
Electricity	30,283.9	7,268,124.8
Heating utility	58,849.6	14,123,896.0
Cooling utility	248,632.6	1,551,467.6
**Potential Co-Products and Credits**		
**Co-products**	**Mass flow (kg/hr)**	**Cost (US$/yr)**
NG	3,768.9	7,839,382.5
Ethane	1,405.9	1,912,089.9
Ethylene	1,738.1	8,064,569.1
Propane	1,617.9	4,271,330.4
Propylene	2,399.7	15,934,192.2
Butane	1,044.0	3,006,589.9
Butene	913.8	9,284,373.8
Light Naphtha	3,573.2	10,862,405.1
Subtotal		61,174,933.0
**Fixed operating costs**		
**Section**	**Calculation method**	**Cost (US$M/yr)**
DAC	3% of CAPEX	27.11
CO_2-to-SAF	3% of CAPEX	21.09
Solar calcination	3% of CAPEX	13.53

Supplementary Table 20: Summary of annual operational expenditure at base case.

Process Section	Cost category (US$M/yr)							
	Fixed cost	Sorbent	Water	Catalyst	Utility	Hydrogen	Co-products	Subtotal
DAC	27.11	9.79	4.13	-	2.85	-	-	43.88
Solar calcination	13.53	-	-	-	-	141.44	-	154.97
CO_2-to-SAF	21.09	-	-	7.96	22.94	160.68	-61.17	151.49
Subtotal	61.72	9.79	4.13	7.96	25.80	302.12	-61.17	350.34

Supplementary Table 21: MSP of SAF at base case.

Process Section	Cost category (US$/kg SAF)									
	Fixed cost	Sorbent	Water	Catalyst	Utility	Hydrogen	Co-products	Capital charge	Land cost	Subtotal
DAC	0.22	0.08	0.03	-	0.02	-	-	0.78	-	1.13
Solar calcination	0.11	-	-	-	-	1.15	-	0.39	-	1.64
CO_2-to-SAF	0.17	-	-	0.06	0.19	1.30	-0.50	0.60	-	1.83
Subtotal	0.50	0.08	0.03	0.06	0.21	2.45	-0.50	1.77	0.02	4.62

Supplementary Table 22: LCOD at base case.

Process Section	Cost category (US$/t CO_2)										
	Fixed cost	Sorbent	Water	Catalyst	Utility	Hydrogen	Co-products	Capital charge	Land cost	SAF avenue	Subtotal
DAC	28.37	10.25	4.32	-	2.99	-	-	100.31	-	-	146.24
Solar calcination	14.61	-	-	-	-	148.04	-	50.07	-	-	212.26
CO_2-to-SAF	22.07	-	-	8.33	24.01	168.17	-64.03	78.04	-	-314.84	-78.24
Subtotal	64.60	10.25	4.32	8.33	27.00	316.21	-64.03	228.42	2.35	-314.84	282.61

Supplementary Table 23: Application and price of co-products[24].

Co-products	Applications	Price (US$/kg)
Natural gas	Fuel	0.26
Ethane	Refrigerant	0.17
Ethylene	Plastics	0.58
Propane	Fuel	0.33
Propylene	Plastics	0.83
Butane	Fuel	0.36
Butene	Plastics	1.27
Light naphtha (C_5-C_7)	Fuel	0.38

Supplementary Table 24: Historical price of jet fuel and sustainable aviation fuel[26].

Price Year	Jet Fuel (US$/t)	SAF (US$/t)
2019	628	NA
2020	365	1412
2021	613	2140
2022	1094	2437

Supplementary Table 25: Technical parameters of CSP.

Parameters	Value	Units	Comments
Average $CaCO_3$ flow rate to the solar calciner	285.3	t_{CaCO3}/hr	Calculated from the process model
Solar power	712.97	MW_{th}	Calculated from the process model
Single heliostat geometry	12.2 x 12.2	m	Adopt design from the CSP plant
Size of the single solar calciner	39.61	MW_{th}	Calculated from the process model
Scaling factor of the solar calciner	13.35	NA	Determined from solar calciner scaling
Hydrogen fluidisation flow rate in a single solar calciner	1.35	t_{H2}/hr	Determined from solar calciner scaling
Number of solar CSP plants	18	NA	Determined from solar calciner scaling and process model
Total solar field area	98.79	acres	Calculated from SAM
Non-solar field land area	45	acres	Obtained from SAM
DNI	2516.2	kWh/m^2/yr	Obtained from Global Solar Atlas
Solar multiple	2.4	NA	Calculated from the process model
Thermal efficiency of solar calciner	60	%	Assumed based on literature[27]

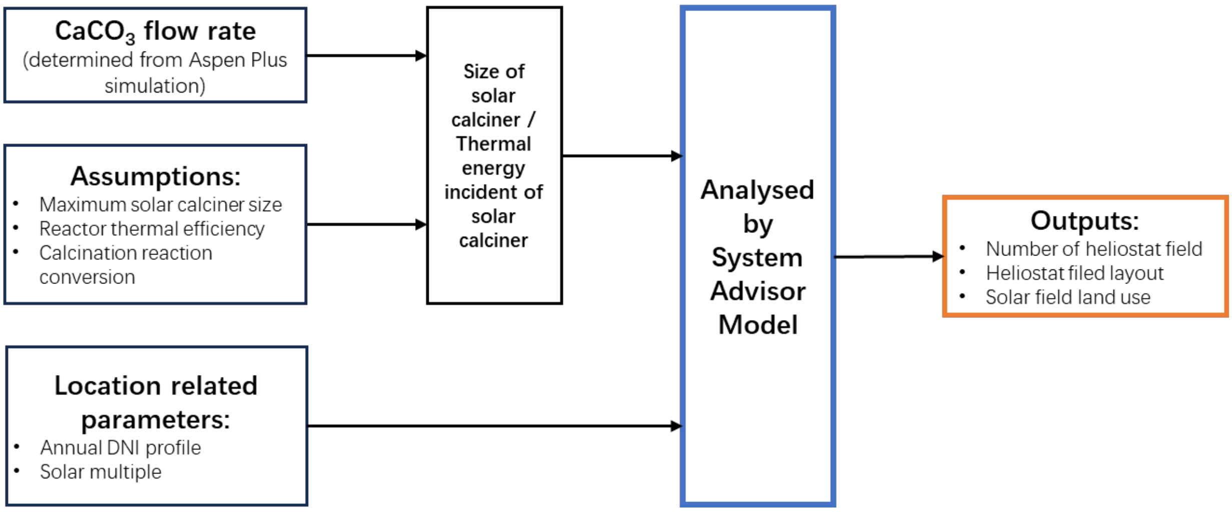

Supplementary Figure 16: Principle of SAM analysis.

The System Advisor Model (SAM) is a tool for designing and optimizing heliostat fields for solar towers based on an annual performance calculation. Based on the selected locations[29], the SAM is used to calculate the requirement of heliostat field area and land area. The imported weather conditions (e.g., annual DNI and wind speed) are from NREL's National Solar Radiation Database (NSRD)[28].

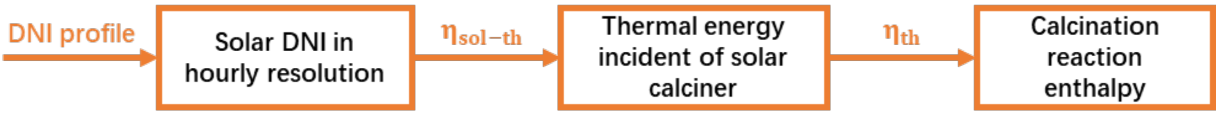

Supplementary Figure 17: Energy flow diagram of solar calcination.

Solar-to-thermal energy efficiency ($\eta_{sloar-th}$), which is defined as the ratio of the solar calciner's thermal energy input to the solar beam irradiation in the solar field. This value is automatically calculated by SAM.

Thermal efficiency (η_{th}), which is defined as the ratio of calcination reaction enthalpy to the solar calciner's thermal energy input. This value is assumed as 60% referring to the literature[27].

Supplementary Table 26: Five selected CSP project locations[28] used for geographical analysis.

Project	Country	Longitude (deg)	Latitude (deg)	Averaged T (℃)	Relative Humidity (%)	Capacity	Technology	SM	Status
Crescent Dunes	USA	39.17	-119.78	12.5	42.7	110 MW	Solar power tower	3	Operational since 2015
Cerro Dominador	Chile	-22.46	-68.93	15.7	17.6	110 MW	Solar power tower	2.4	Operational since 2021
Planta Solar 20	Spain	37.45	-6.26	19.1	58.9	20 MW	Solar power tower	3	Operational since 2009
Redstone CSP Project	South Africa	-28.31	23.38	18.2	28.8	100 MW	Solar power tower	2.7	Operational since 2023
Shouhang Dunhuang Phase II	China	40.05	94.42	12.0	22.5	100 MW	Solar power tower	3	Operational since 2018

Supplementary Table 27: Regional hydrogen cost[29-31], PV cost[32] and WACC[33].

Country	Hydrogen production technology and cost (US$/kg)				PV electricity price (US$/MWh)	WACC (%)
	AE	PEM	SOEC	SMR with CCS		
USA	2.5 - 4.0	3.0 - 5.0	4.5 - 6.5	1.5 - 2.0	41	5.10
Chile	1.8 - 3.2	2.5 - 4.0	3.8 - 5.8	1.2 - 1.7	32	9.20
Spain	2.2 - 3.7	2.8 - 4.3	4.0 - 6.0	1.4 - 1.9	36	4.20
South Africa	2.3 - 3.8	2.9 - 4.4	4.2 - 6.2	1.3 - 1.8	42	11.80
China	2.0 - 3.5	2.7 - 4.2	3.9 - 5.9	1.1 - 1.6	23	6.60

Supplementary Note 7: Sensitivity analysis

All process analyses of air contactors are under the same liquid solvent composition (2 M K^+, 1.1 M OH^-, 0.45 M CO_3^{2-}) because the rate-based model was specifically developed under this circumstance. The process analysis was carried out for CO_2 concentration in the air, air velocity (V_{air}), air travel distance (ATD) and the ratio of the mass flow rate of liquid solvent over the mass flow rate of air (L/G ratio).

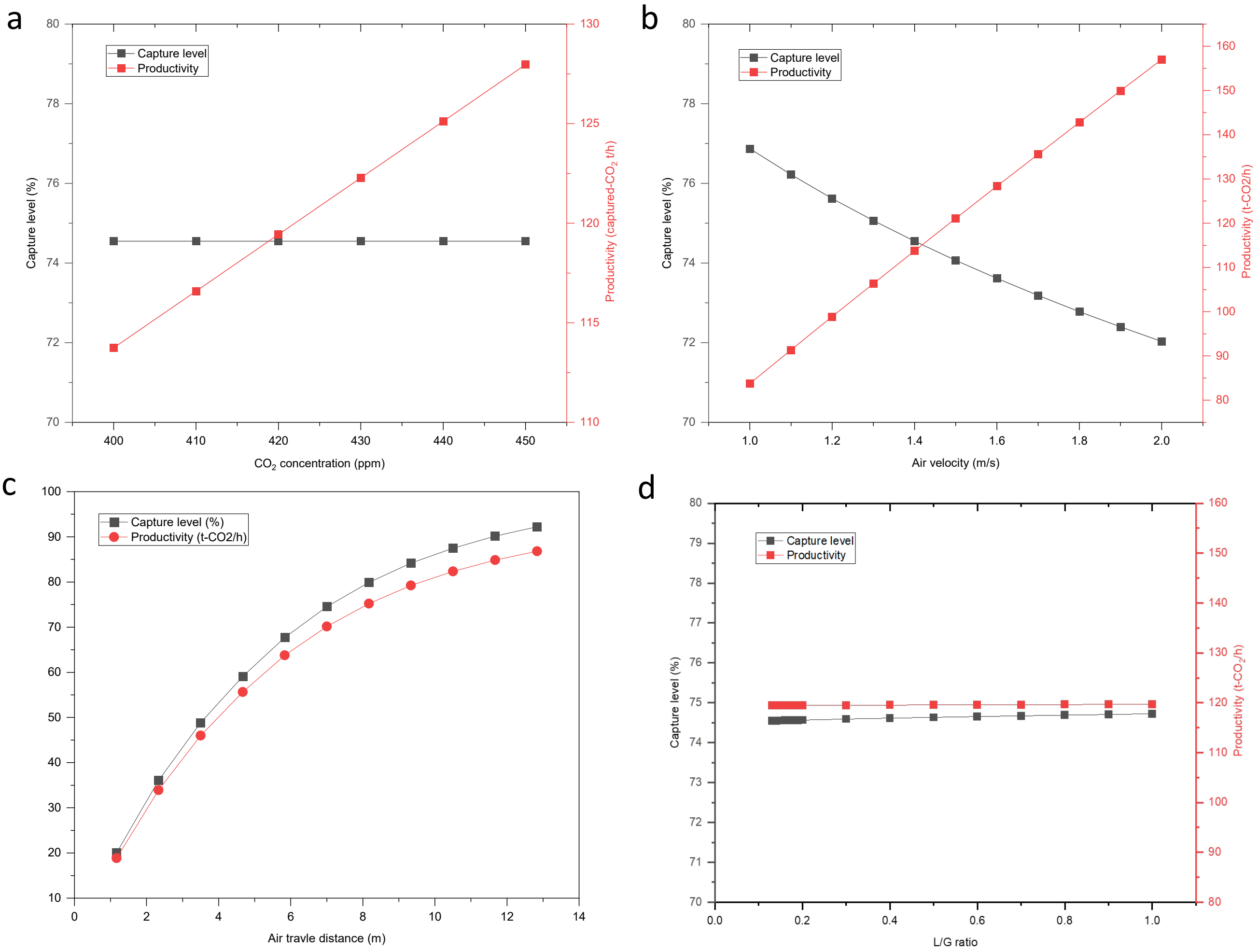

Supplementary Figure 18: Impact of operating and design variables on capture level and productivity. Variables are (a) CO_2 concentration in the air, (b) air velocity, (c) air travel distance and (d) L/G ratio.

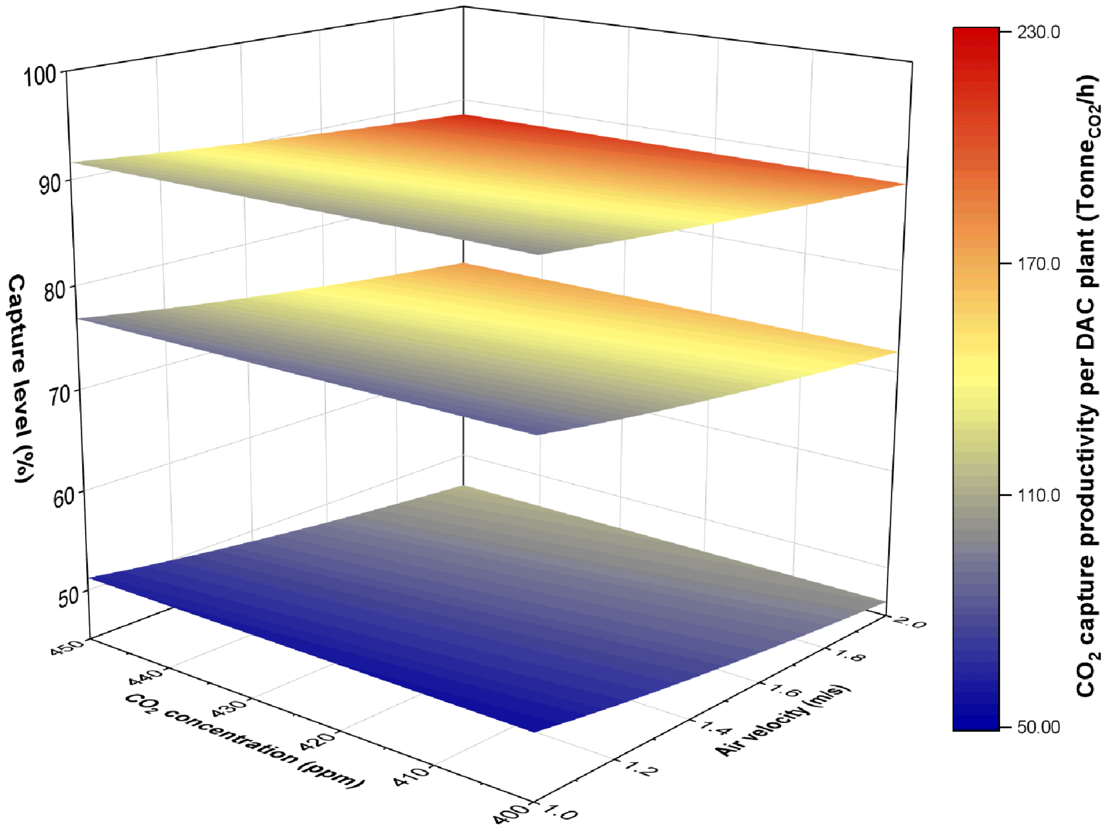

Supplementary Figure 19: Map of DAC CO_2 capture productivity. CO_2 productivity as a function of CO_2 concentration in the air, air velocity, and air travel distance (ATD). Three coloured layers represent capture level at around 50%, 75% and 90% with 3 segments (3.5 m ATD), 6 segments (7 m ATD) and 10 segments (11.7 m ATD) Brentwood XF12560 packing, respectively.

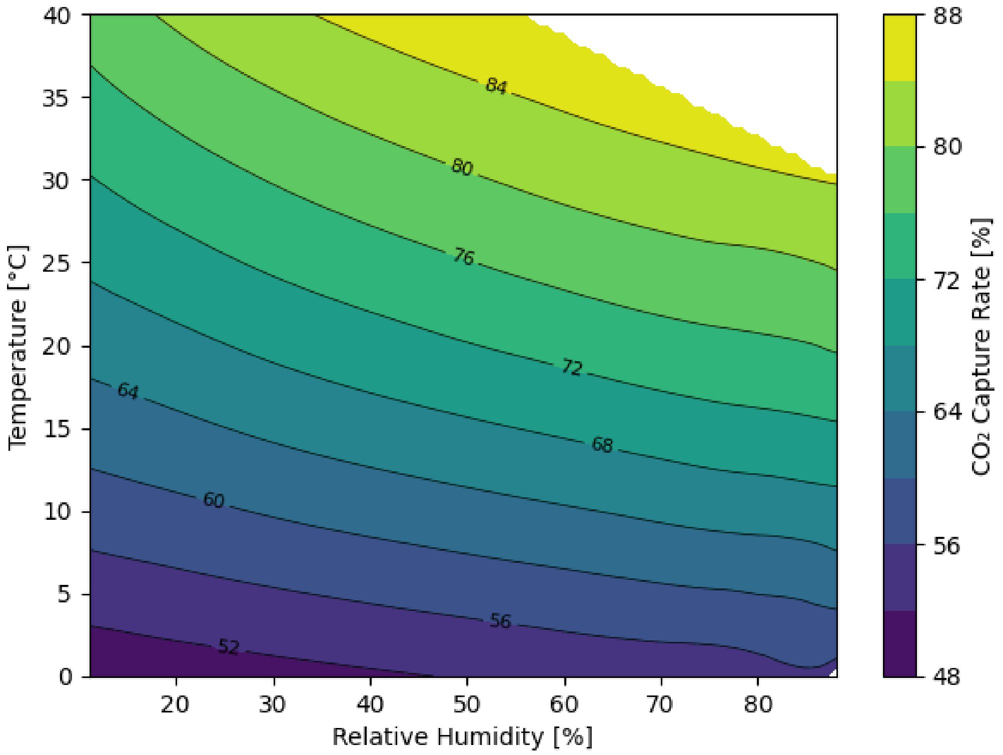

Supplementary Figure 20: CO_2 capture rate at different relative humidity and temperature ranges. White-coloured area represents unreachable air conditions.

Supplementary Table 28: Sensitivity analysis of parameters for DAC material requirement and electricity consumption under different operating conditions.

Parameter	Pr	conc	V_{air}	ATD	CL	F_{air}	F_{KOH}	Re_{KOH}	$F_{Ca(OH)2}$	F_{water}	Fan ΔP	E_{fan}	E_{pump}	E_{DAC}	F_{CaCO3}
Unit	t-CO_2/h	ppm	m/s	m	%	kt/h	kt/h	%	t/h	t	Pa	kWh/t-CO_2	kWh/t-CO_2	kWh/t-CO_2	t/h
**Base case**	119.4	420	1.4	7	74.5	251.0	32.6	99.7	716.5	516.1	85.7	57.4	42.1	99.5	238.7
**400 ppm**	113.7	400	1.4	7	74.5	251.0	32.6	99.7	682.4	489.7	85.7	60.3	44.2	104.5	271.7
**450 ppm**	128.0	450	1.4	7	72.0	251.0	32.6	99.7	767.7	555.8	85.7	53.6	39.3	92.9	305.7
**1m/s V_{air}**	88.0	420	1	7	76.9	179.3	23.3	99.7	527.7	369.8	43.0	27.9	40.9	68.8	210.1
**2m/s V_{air}**	164.9	420	2	7	74.5	358.6	46.6	99.7	989.1	727.4	181.4	125.8	43.6	169.4	393.8
**3.5m ATD**	78.3	420	1.4	3.5	48.9	251.0	16.3	99.7	469.6	324.7	51.0	52.1	32.1	84.2	187.0
**11.7m ATD**	144.4	420	1.4	11.7	90.1	251.0	54.5	99.7	866.4	632.3	126.1	69.8	58.2	128.1	345.0

Under different operating conditions, the L/G ratio and packing wetting condition were maintained the same. The airflow rate was based on CO_2 concentration in the air (conc) and air velocity (V_{air}). Thereby, the KOH solvent flow rate (F_{KOH}) was determined by the inlet air flow rate (F_{air}). The mass flow rate of $Ca(OH)_2$ slurry ($F_{Ca(OH)2}$) depends on how much CO_2 is captured by the air contactor for subsequent decomposition in the pellet reactor with calcium ions. The water flow rate (F_{water}) is based on the mass balance of the process model.

Electricity demand for DAC is influenced by a combination of parameters ($V_{air,}$ ATD and conc). In the optimistic scenario, higher V_{air} increases the volume of air processed and thus requires higher solvent flow rates and greater fan and pump power. Longer ATD requires more liquid to wet the packing column, increasing pump energy. Higher conc reduces electricity demand due to improved capture productivity (Pr). Conversely, in the pessimistic scenarios, lower V_{air} and shorter ATD result in reduced airflow and solvent use, which decreases fan and pump electricity demand. While for the lower conc case, the system requires more electricity.

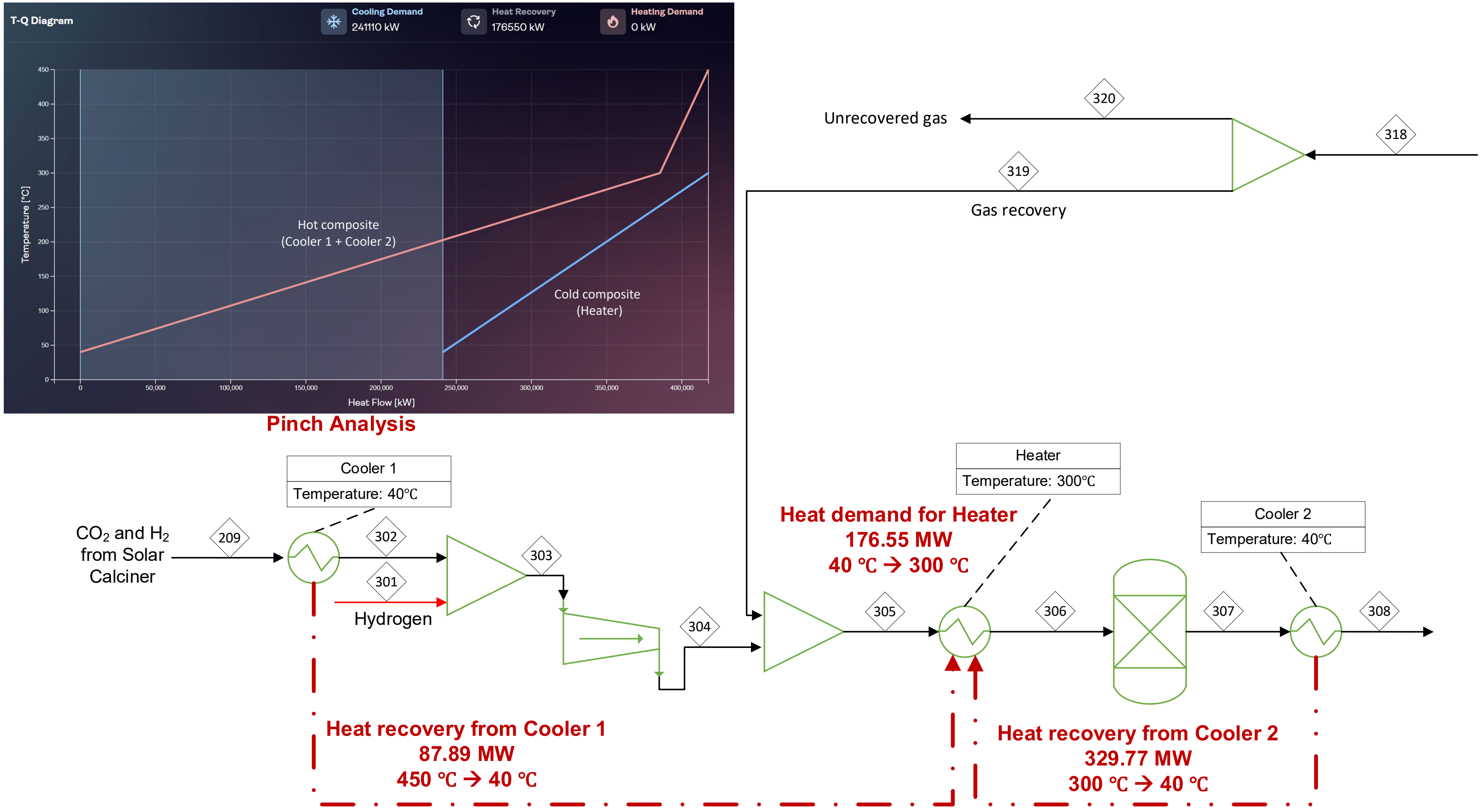

Supplementary Figure 21: Potential heat integration in the CO_2-to-SAF section at base case scenario.

Using a heat recovery strategy and pinch analysis, the heat from 450°C mixed gas (Stream 209) and 300°C syncrude (Stream 307) can be recovered to preheat the 40°C gas (Stream 305). This heat recovery results in the savings of 176.55 MW for heating in the CO_2-to-SAF section.

Supplementary Table 29: Sensitivity analysis summary of MSP, related to Fig. 4d.

Sensitivity parameter	Sensitivity parameter ranges				MSP (US$/kg)		%MSP difference (from base case)		Justification and parameter range
	Unit	Optimistic case	Base case	Pessimistic case	Optimistic MSP	Pessimistic MSP	Optimistic%	Pessimistic%	
CO_2 concentration	ppm	450	420	400	4.58	4.65	-0.9	0.6	Ranges of CO_2 concentration in the air[34]. 450 ppm represents the global warming 2℃ target.
Air velocity	m/s	2	1.4	1	4.42	4.79	-4.5	3.6	Carbon Engineering's pilot plant operation condition[2]
Air travel distance	m	11.7	7	3.5	4.51	4.85	-2.5	4.8	Based on the process model, controlling the air travel distance, the CO_2 capture rate was modelled at around 50%, 75% and 90%
Temperature	℃	30	21	0	4.57	4.85	-1.2	5.0	Based on process model and ref[35]
Relative humidity	%	80	64	20	4.62	4.70	-0.1	1.6	Based on process model and ref[35]
Thermal efficiency of solar calciner	%	80%	60%	40%	4.50	4.86	-2.7	5.1	Referring to CSP plant[27,36]
Solar multiple	NA	2.5	3	3.5	4.45	4.78	-3.8	3.3	Referring to the CSP plant[28,37]
CAPEX of CSP	US$M	189.5	378.9	568.4	4.38	4.87	-5.4	5.4	$\pm$50% based on process model
Gas recovery ratio	%	99	90	80	3.96	5.37	-14.4	16.1	Based on process model
Hydrogen production cost	US$/kg	1	2	3	3.50	5.75	-24.3	24.3	Baseline is at US$2/kg which is the short-term USA target[38]. US$1/kg is the long-term target. Sensitivity analysis investigates $\pm$50% of hydrogen production cost.
Plant lifetime	year	40	30	20	4.56	4.82	-1.4	4.1	Baseline is at 30 years, and sensitivity analysis investigates $\pm$10 years of plant lifetime[39]
WACC	%	5	10	15	3.93	5.40	-14.9	16.8	Baseline is at 10%[40], and sensitivity analysis investigates $\pm$50% of WACC[33].
PV electricity price	US$/MWh	10	30	60	4.49	4.82	-2.8	4.2	PV cost based on ref[32]
Land cost	US$/$m^2$	1.24	2.47	49.42	4.62	4.97	-0.2	7.5	Baseline land cost is based on ref[22]. The optimistic case uses -50% cost while the pessimistic cost is based on ref[41].

Supplementary Table 30: Sensitivity analysis summary of LCOD, related to Fig. 4e.

Sensitivity parameter	Sensitivity parameter ranges				LCOD (US$/t CO_2)		%LCOD difference (from base case)		Justification and parameter range
	Unit	Optimistic case	Base case	Pessimistic case	Optimistic LCOD	Pessimistic LCOD	Optimistic%	Pessimistic%	
CO_2 concentration	ppm	450	420	400	277.0	286.2	-2.0	1.3	Ranges of CO_2 concentration in the air[34]. 450 ppm represents the global warming 2℃ target.
Air velocity	m/s	2	1.4	1	255.8	303.9	-9.5	7.6	Carbon Engineering's pilot plant operation condition[2]
Air travel distance	m	11.7	7	3.5	267.4	311.5	-5.4	10.2	Based on the process model, controlling the air travel distance, the CO_2 capture rate was modelled at around 50%, 75% and 90%
Temperature	℃	30	21	0	275.4	313.3	-2.5	10.5	Based on process model and ref[35]
Relative humidity	%	80	64	20	281.8	292.4	-0.3	3.5	Based on process model and ref[35]
Thermal efficiency of solar calciner	%	80%	60%	40%	266.3	313.3	-5.8	10.9	Referring to CSP plant[27,36]
Solar multiple	NA	2.5	3	3.5	260.1	302.4	-8.0	7.0	Referring to the CSP plant[28,37]
CAPEX of CSP	US$M	189.5	378.9	568.4	250.5	314.7	-11.4	11.4	±50% based on process model
Gas recovery ratio	%	99	90	80	226.2	330.8	-20.0	17.0	Based on process model
SAF market price	US$/kg	1.24	2.47	3.71	125.2	440.0	-55.7	55.7	Baseline SAF market price uses 2022 data[26]. Sensitivity analysis investigates ±50% of SAF market price.
Hydrogen production cost	US$/kg	1	2	3	137.6	427.7	-51.3	51.3	Baseline is at US$2/kg which is the short-term USA target[38]. US$1/kg is the long-term target. Sensitivity analysis investigates ±50% of hydrogen production cost.
Plant lifetime	Year	40	30	20	274.3	307.4	-2.9	8.8	Baseline is at 30 years, and sensitivity analysis investigates ±10 years of plant lifetime[39]
WACC	%	5	10	15	193.4	383.2	-31.6	35.6	Baseline is at 10%[40], and sensitivity analysis investigates ±50% of WACC[33].
PV electricity price	US$/MWh	10	30	60	265.4	308.0	-6.0	9.0	PV cost based on ref[32]
Land cost	US$/$m^2$	1.24	2.47	49.42	281.4	327.2	-0.4	15.8	Baseline land cost is based on ref[22]. The optimistic case uses -50% cost while the pessimistic cost is based on ref[41].

References

1 Sabatino, F. *et al.* A comparative energy and costs assessment and optimization for direct air capture technologies. *Joule* **5**, 2047-2076 (2021).

2 Keith, D. W., Holmes, G., Angelo, D. S. & Heidel, K. A process for capturing CO_2 from the atmosphere. *Joule* **2**, 1573-1594 (2018).

3 Holmes, G. J. *A carbon dioxide absorption performance evaluation for capture from ambient air*, University of Calgary, (2010).

4 CoolingTowerDepot®. *The depot of all cooling towers*, <http://www.coolingtowerdepot.com/content/cooling-towers> (2023).

5 Fair, J. R., Seibert, A. F., Behrens, M., Saraber, P. & Olujic, Z. Structured packing performance experimental evaluation of two predictive models. *Industrial & Engineering Chemistry Research* **39**, 1788-1796 (2000).

6 Heidel, K., Keith, D., Singh, A. & Holmes, G. Process design and costing of an air-contactor for air-capture. *Energy Procedia* **4**, 2861-2868 (2011).

7 Holmes, G. & Keith, D. W. An air–liquid contactor for large-scale capture of CO_2 from air. *Philosophical Transactions of the Royal Society A: Mathematical, Physical and Engineering Sciences* **370**, 4380-4403 (2012).

8 de Jonge, M. M., Daemen, J., Loriaux, J. M., Steinmann, Z. J. & Huijbregts, M. A. Life cycle carbon efficiency of Direct Air Capture systems with strong hydroxide sorbents. *International Journal of Greenhouse Gas Control* **80**, 25-31 (2019).

9 Holmes, G. *et al.* Outdoor prototype results for direct atmospheric capture of carbon dioxide. *Energy Procedia* **37**, 6079-6095 (2013).

10 Madhu, K., Pauliuk, S., Dhathri, S. & Creutzig, F. Understanding environmental trade-offs and resource demand of direct air capture technologies through comparative life-cycle assessment. *Nature Energy* **6**, 1035-1044 (2021).

11 Heidel, K. & Rossi, R. United States Patent application: 0170327421. *High Temperature Hydrator (A1). Filed May* **10** (2017).

12 Robie, R. A. & Hemingway, B. S. *Thermodynamic properties of minerals and related substances at 298.15 K and 1 bar (105 Pascals) pressure and at higher temperatures*. Vol. 2131 (US Government Printing Office, 1995).

13 Hilsenrath, J. *Tables of thermal properties of gases: comprising tables of thermodynamic and transport properties of air, argon, carbon dioxide, carbon monoxide, hydrogen, nitrogen, oxygen, and steam*. Vol. 564 (US Department of Commerce, National Bureau of Standards, 1955).

14 Esence, T., Guillot, E., Tessonneaud, M., Sans, J.-L. & Flamant, G. Solar calcination at pilot scale in a continuous flow multistage horizontal fluidized bed. *Solar Energy* **207**, 367-378 (2020).

15 Yao, B. *et al.* Transforming carbon dioxide into jet fuel using an organic combustion-synthesized Fe-Mn-K catalyst. *Nature Communications* **11**, 6395 (2020).

16 Zhang, L. *et al.* Direct conversion of CO_2 to a jet fuel over CoFe alloy catalysts. *The Innovation* **2**, 100170 (2021).

17 Kamkeng, A. D. & Wang, M. Technical analysis of the modified Fischer-Tropsch synthesis process for direct CO_2 conversion into gasoline fuel: Performance improvement via ex-situ water removal. *Chemical Engineering Journal* **462**, 142048 (2023).

18 Todic, B. *et al.* Kinetic model of Fischer–Tropsch synthesis in a slurry reactor on Co–Re/Al_2O_3 catalyst. *Industrial & Engineering Chemistry Research* **52**, 669-679 (2013).

19 Donnelly, T. J., Yates, I. C. & Satterfield, C. N. Analysis and prediction of product distributions of the Fischer-Tropsch synthesis. *Energy & Fuels* **2**, 734-739 (1988).

20 Kelkar, V. V. & Ng, K. M. Development of fluidized catalytic reactors: Screening and scale-up. *AIChE journal* **48**, 1498-1518 (2002).

21 Prats-Salvado, E., Jagtap, N., Monnerie, N. & Sattler, C. Solar-Powered Direct Air Capture: Techno-Economic and Environmental Assessment. *Environmental Science & Technology* **58**, 2282-2292 (2024).

22 Buck, R. & Sment, J. Techno-economic analysis of multi-tower solar particle power plants. *Solar Energy* **254**, 112-122 (2023).

23 Zang, G., Sun, P., Elgowainy, A. A., Bafana, A. & Wang, M. Performance and cost analysis of liquid fuel production from H_2 and CO_2 based on the Fischer-Tropsch process. *Journal of CO_2 Utilization* **46**, 101459 (2021).

24 Yadav, G. *et al.* Techno-economic analysis and life cycle assessment for catalytic fast pyrolysis of mixed plastic waste. *Energy & Environmental Science* **16**, 3638-3653 (2023).

25 Liu, C. M., Sandhu, N. K., McCoy, S. T. & Bergerson, J. A. A life cycle assessment of greenhouse gas emissions from direct air capture and Fischer–Tropsch fuel production. *Sustainable Energy & Fuels* **4**, 3129-3142 (2020).

26 IATA. *IATA Economics*, < https://www.iata.org/en/publications/economics/> (2024).

27 Bellos, E. Progress in beam-down solar concentrating systems. *Progress in Energy and Combustion Science* **97**, 101085 (2023).

28 NREL. *Power Tower Projects*, <https://solarpaces.nrel.gov/by-technology/power-tower> (2024).

29 IEA. *Global Hydrogen Review 2023 – Analysis*, <https://www.iea.org/reports/global-hydrogen-review-2023> (2023).

30 McKinsey & Company. *Global Energy Perspective 2023: Hydrogen outlook*, <https://www.mckinsey.com/industries/oil-and-gas/our-insights/global-energy-perspective-2023-hydrogen-outlook> (2024).

31 Hydrogen Council. *Hydrogen Insights 2023*, <https://hydrogencouncil.com/en/hydrogen-insights-2023/> (2024).

32 IEA. *Global Hydrogen Review 2023: Assumptions Annex*, <https://iea.blob.core.windows.net/assets/101dd112-b72b-4a74-82f1-de7fea6ae48e/GlobalHydrogenReview2023_AssumptionsAnnex.pdf> (2023).

33 Ameli, N. *et al.* Higher cost of finance exacerbates a climate investment trap in developing economies. *Nature Communications* **12**, 1-12 (2021).

34 NASA. *Carbon dioxide concentration*, <https://climate.nasa.gov/vital-signs/carbon-dioxide/?intent=121> (2024).

35 An, K., Farooqui, A. & McCoy, S. T. The impact of climate on solvent-based direct air capture systems. *Applied Energy* **325**, 119895 (2022).

36 Zhao, J. *et al.* Particle-based high-temperature thermochemical energy storage reactors. *Progress in Energy and Combustion Science* **102**, 101143 (2024).

37 NREL. *NSRDB: National Solar Radiation Database*, <https://nsrdb.nrel.gov/data-viewer> (2024).

38 Department of Energy. *U.S. National Clean Hydrogen Strategy and Roadmap*, <https://www.hydrogen.energy.gov/docs/hydrogenprogramlibraries/pdfs/us-national-clean-hydrogen-strategy-roadmap.pdf?sfvrsn=c425b44f_5> (2023).

39 Fasihi, M., Efimova, O. & Breyer, C. Techno-economic assessment of CO_2 direct air capture plants. *Journal of Cleaner Production* **224**, 957-980 (2019).

40 IEAGHG. *Global Assessment of Direct Air Capture Costs*, <https://publications.ieaghg.org/technicalreports/2021-05%20Global%20Assessment%20of%20Direct%20Air%20Capture%20Costs.pdf> (2021).

41 landsearch. *Price of Land per Acre by State - LandSearch*, <https://www.landsearch.com/price> (2024).
